# Three Prime Repair Exonuclease 1 (TREX1) expression correlates with cervical cancer cells growth *in vitro* and disease progression *in vivo*

**DOI:** 10.1038/s41598-018-37064-x

**Published:** 2019-01-23

**Authors:** Bruna Prati, Walason da Silva Abjaude, Lara Termini, Mirian Morale, Suellen Herbster, Adhemar Longatto-Filho, Rafaella Almeida Lima Nunes, Lizeth Carolina Córdoba Camacho, Silvia Helena Rabelo-Santos, Luiz Carlos Zeferino, Francisco Aguayo, Enrique Boccardo

**Affiliations:** 1 Department of Microbiology, Institute of Biomedical Sciences, University of São Paulo (USP), Av. Prof. Lineu Prestes 1374, 05508-900 São Paulo, SP Brazil; 2 Centro de Investigação Translacional em Oncologia (LIM24), Instituto do Câncer do Estado de São Paulo (ICESP), São Paulo, Brazil; 3 Department of Biochemistry, Institute of Chemistry, USP, São Paulo, Brazil; 4 Laboratory of Medical Investigation (LIM 14), Department of Pathology, School of Medicine, USP, Av. Dr. Arnaldo 455, São Paulo, 01246-903 Brazil; 5 Life and Health Sciences Research Institute, School of Health Sciences, ICVS/3B’s - PT Government Associate Laboratory, University of Minho, Braga, Guimarães Portugal; 6 Molecular Oncology Research Center, Barretos Cancer Hospital, Pio XII Foundation, Barretos, Rua Antenor Duarte Villela, 1331, Barretos, 14784-400 Brazil; 7 School of Pharmacy, Federal University of Goiás, Avenida Universitária, 74605-220 Goiânia, GO Brazil; 8 School of Medical Sciences, State University of Campinas (UNICAMP), Rua Alexander Fleming 101, 13083-881 Campinas, SP Brazil; 9 Basic and Clinical Oncology Department, Faculty of Medicine, University of Chile, Santiago, Chile; 10 Advanced Center for Chronic Diseases (ACCDiS), Pontificia Universidad Católica de Chile, Santiago, Chile; 11 Laboratório de Oncologia Experimental, Departamento de Radiologia, Faculdade de Medicina, USP, São Paulo, SP Brazil; 12 Present Address: Centro de Investigação Translacional em Oncologia, ICESP, São Paulo, SP Brazil

## Abstract

Alterations in specific DNA damage repair mechanisms in the presence of human papillomavirus (HPV) infection have been described in different experimental models. However, the global effect of HPV on the expression of genes involved in these pathways has not been analyzed in detail. In the present study, we compared the expression profile of 135 genes involved in DNA damage repair among primary human keratinocytes (PHK), HPV-positive (SiHa and HeLa) and HPV-negative (C33A) cervical cancer derived cell lines. We identified 9 genes which expression pattern distinguishes HPV-positive tumor cell lines from C33A. Moreover, we observed that Three Prime Repair Exonuclease 1 (TREX1) expression is upregulated exclusively in HPV-transformed cell lines and PHK expressing HPV16 E6 and E7 oncogenes. We demonstrated that TREX1 silencing greatly affects tumor cells clonogenic and anchorage independent growth potential. We showed that this effect is associated with p53 upregulation, accumulation of subG1 cells, and requires the expression of E7 from high-risk HPV types. Finally, we observed an increase in TREX1 levels in precancerous lesions, squamous carcinomas and adenocarcinomas clinical samples. Altogether, our results indicate that TREX1 upregulation is important for cervical tumor cells growth and may contribute with tumor establishment and progression.

## Introduction

Human papillomaviruses (HPV) are small, non-enveloped DNA viruses which belong to the *Papillomaviridae* family with marked tropism for stratified epithelia at specific anatomic sites^1,2^. Approximately 40 HPV types infect the anogenital tract mucosa and are classified as low- or high- oncogenic risk types according to the associated lesions. Low-risk HPV types (i.e. HPV6 and HPV11) are associated with hyperproliferative lesions with low tendency to malignant progression. On the other hand, high-risk (HR) HPV types namely, HPV16, -18, -31, -33, -35, -39, -45, -51, -52, -56, -58 and -59 are classified as type I carcinogens by the International Agency for Research on Cancer (IARC) due to their etiological association with cervical cancer. Besides, high-risk HPV types are associated with a significant fraction of vulvar, vaginal, anal, penile and oropharyngeal carcinomas.

A hallmark of HPV associated tumors is the continuous expression of viral E6 and E7 oncoproteins. The main characteristic of HR-HPV E6 and E7 is their ability to mediate p53 and pRb degradation by proteasomal machinery, respectively^3–9^. Besides, these viral proteins target other cellular factors that affect keratinocyte’s proliferation, lifespan, differentiation and survival. Consequently, HPV oncoproteins expression promote genome instability and accumulation of mitotic defects in infected cells contributing with cell transformation and tumor progression^10–15^.

In addition to the continuous expression of viral oncogenes, accumulation of additional genetic alterations by host cell is required for the development of a malignant tumor. In fact, a complex pattern of structural and numerical chromosomal alterations are generally observed in pre-malignant lesions of the uterine cervix. Gains in 1, 3q, 5p, 6p, 7, 8q, 9q, 16q and 20, as well as losses in 2q, 3p, 4q, 6q, 11q, 13q, 16, 17 have been associated with HPV presence^16–22^. Besides, genomic alterations and amplification of certain genes have been observed in other HPV-positive carcinomas^23–25^.

Alterations in DNA damage repair systems due to HPV presence have been described in different experimental models. For instance, deficiencies in the nucleotide excision repair (NER) mechanism were observed in HPV16-immortalized oral keratinocytes^26^. The expression of HPV16 E6 has been associated with defects in both global and transcription-coupled nucleotide excision repair (GNER and TCNER, respectively), reduced ability to remove thymine dimers induced by UV, downregulation of double strand breaks repair and degradation of O^6^-methylguanine-DNA methyltransferase^27–29^. Besides, the presence of this viral protein abrogates p53R2 induction and p53-mediated response to DNA damage and oxidative stress^30^. Finally, it has been reported that fibroblasts expressing HPV16 E7 are deficient in GNER^27^ and that sustained expression of HR-HPV E6 and E7 oncoproteins induces DNA breaks and increases the integration rate of foreign DNA in host cells^6,31^.

These observations underscore the importance of DNA repair mechanisms in HPV-mediated pathogenesis. However, the presence of global alterations in these pathways in HPV-transformed cells has not been addressed. In the present study, we compared the expression profile of 135 genes involved in different DNA damage repair pathways among primary human keratinocytes (PHK) and HPV-positive (SiHa and HeLa) and HPV-negative (C33A) cervical cancer derived cell lines. We observed that tumor derived cell lines exhibit a high number of differentially expressed genes when compared to normal PHK. Interestingly, we showed that the levels of the Three Prime Repair Exonuclease 1 (TREX1) were upregulated exclusively in monolayer and organotypic cultures of cells expressing HPV oncogenes. Besides, we provided evidence that TREX1 silencing inhibits tumor cells growth by inducing p53 upregulation and accumulation of SubG1 cells. Furthermore, we showed that these effects required the expression of E7 from high-risk HPV types. Importantly, using human cervical tissues samples we demonstrated that TREX1 levels are low in normal cervical epithelium but increase in precancerous lesions, squamous carcinoma and adenocarcinoma. This observation was further confirmed in four cervical cancer expressions array series from Gene Expression Omnibus (GEO) dataset. Altogether, our results reveal the presence of significant changes in the expression of genes involved in DNA damage repair pathways in cervical cancer derived cell lines. Besides, our functional analyses suggest that TREX1 upregulation is important for tumor cells survival, tumorigenic potential and tumor establishment/progression.

## Results

### Cervical cancer derived cell lines exhibit extensive alterations in the expression of genes involved in DNA damage repair

In order to identify alterations in DNA damage repair pathways in cervical cancer derived cell lines we analyzed the expression of 135 genes involved in DNA damage signaling and repair using commercial RT-PCR arrays. Total RNA samples from SiHa (HPV16), HeLa (HPV18) and C33A (HPV-negative) cell lines were analyzed using primary human keratinocytes as the reference group. Gene expression results presented are the average from three independent experiments for each cell line. We observed that the majority of genes were differentially expressed between any tumor cell line and normal PHKs with statistical significance (p-value ≤ 0.05) (Supplementary Fig. 1). Among the differentially expressed genes, we selected those that were up- or downregulated by 2,0-fold between PHK and the three tumor cell lines. The list of 18 genes identified and their attributed functions is presented in Table 1.

**Table 1:** List of genes differentially expressed between cervical cancer derived cell lines and PHK.

*Gene*	*Description*	C33A		SiHa		HeLa	
		Fold (*)	*p-value*	*Fold*	*p-value*	*Fold*	*p-value*
BRIP1	BRCA1 interacting protein C-terminal helicase 1	4,20	0,00016	2,39	0,00395	2,07	0,01392
DMC1	DMC1 dosage suppressor of mck1 homolog, meiosis-specific homologous recombination (yeast)	−6,21	0,03704	9,06	0,00607	−3,02	0,00553
EXO1	Exonuclease 1	6,11	0,00180	5,14	0,00001	3,02	0,00587
FANCG	Fanconi anemia, complementation group G	4,35	0,00098	4,08	0,02178	3,70	0,04354
FEN1	Flap structure-specific endonuclease 1	4,71	0,00912	4,44	0,0247	4,25	0,01481
GTSE1	G-2 and S-phase expressed 1	9,31	0,00035	4,87	0,0002	3,94	0,00044
IGHMBP2	Immunoglobulin mu binding protein 2	4,74	0,0029	9,39	0,01658	3,26	0,00429
LIG1	Ligase I, DNA, ATP-dependent	13,04	0,00053	15,29	0,01269	9,78	0,00048
MAP2K6	Mitogen-activated protein kinase kinase 6	10,81	0,04978	29,68	0,00472	36,53	0,01302
MRE11A	MRE11 meiotic recombination 11 homolog A (S. cerevisiae)	3,59	0,00906	4,76	0,01521	4,17	0,00804
MSH4	MutS homolog 4 (*E. coli*)	17,53	0,0018	−2,99	0,01202	−4,55	0,03382
NTHL1	Nth endonuclease III-like 1 (*E. coli*)	5,13	0,00044	3,63	0,01203	3,40	0,0154
RAD51	RAD51 homolog B (S. cerevisiae)	2,83	0,01412	6,66	0,0025	3,65	0,01687
RAD54L	RAD54-like (S. cerevisiae)	2,28	0,00371	3,43	0,02211	2,61	0,00442
RAD9A	RAD9 homolog A (S. pombe)	6,15	0,00222	12,28	0,01104	4,66	0,00397
RPA1	Replication protein A1, 70 kDa	2,78	0,00021	5,06	0,00641	2,41	0,00443
SMC1A	Structural maintenance of chromosomes 1A	8,03	0,00034	15,54	0,00249	4,68	0,03638
TREX1	Three prime repair exonuclease 1	−2,48	0,00054	4,56	0,00846	2,96	0,00055

*Only genes with fold >2 or fold <−2 were included.

Next, we performed a new analysis comparing the gene expression pattern between the HPV-positive and the HPV-negative (reference group) cervical cancer cell lines. Using this approach we identified 9 genes with fold >2 or fold <−2 and p-value < 0.05, that differentiate HeLa and SiHa cells from the HPV-negative C33A cell line (Table 2). Interestingly, the alteration in the expression pattern of these genes, either up- or downregulation was similar for both HPV-positive cell lines.

**Table 2:** List of genes differentially expressed between HPV-positive cell lines when were compared to C33A.

*Gene*	*Description*	HeLa		SiHa	
		Fold (*)	*p-value*	*Fold*	*p-value*
CHEK2	CHK2 checkpoint homolog (S. pombe)	−12,09	0,00355	−3,04	0,01126
DMC1	DMC1 dosage suppressor of mck1 homolog, meiosis-specific homologous recombination (yeast)	2,06	0,02915	56,26	0,00388
ERCC5	Excision repair cross-complementing rodent repair deficiency, complementation group 5	2,23	0,02225	3,55	0,00323
MSH4	MutS homolog 4 (*E. coli*)	−79,86	0,00146	−52,56	0,00170
NBN	Nibrin	−23,73	0,01229	−2,30	0,01673
NEIL2	Nei endonuclease VIII-like 2 (*E. coli*)	−8,65	0,00415	−5,85	0,00602
OGG1	8-oxoguanine DNA glycosylase	−2,27	0,01522	−2,16	0,00791
TREX1	Three prime repair exonuclease 1	7,33	0,00589	11,29	0,00063
XRCC2	X-ray repair complementing defective repair in Chinese hamster cells 2	−5,51	0,00617	−2,33	0,00663

*Only genes with fold >2 or fold <−2 were included.

### Validation of differentially expressed genes

In order to confirm the results obtained after mRNA level analysis (Supplementary Fig. 1, Tables 1 and 2) some genes were selected for validation at the protein level, namely: *CHEK2* (cell cycle checkpoint kinase 2), *FEN1* (Flap endonuclease), *LIG1* (DNA ligase I), *MAP2K6* (mitogen-activated protein kinase kinase 6), *MRE11A* (MRE11 Homolog A), *PCNA* (proliferating cell nuclear antigen), *PNK* (polynucleotide kinase), *RAD9A* (RAD9 homolog A), *RAD51* (RAD51 homolog), *RPA1* (replication protein A1), *SMC1A* (structural maintenance of chromosomes 1A) and *TREX1* (three prime repair exonuclease 1). For this selection we considered: (a) genes with greater expression level difference when compared to the control group; (b) genes that consistently appeared in the different comparisons performed; (c) data from the literature; and (d) antibody availability.

The levels of these proteins were determined by western blot using total protein extracts obtained from tumor cell lines and PHK transduced with E6 and E7 from HPV11 (low-risk) and HPV16 (high-risk). We observed that LIG1, MRE11A, RAD9 and SMC1A levels were up-regulated in tumor cell lines when compared to normal PHK or to PHK expressing HPV genes (Supplementary Figs 2 and 3). This observation suggests that alterations in these factors expression may play a role during cell transformation or be involved in tumor establishment/progression.

Interestingly, we observed that FEN1, PCNA, RAD51 and RPA1 protein levels were upregulated in cervical cancer derived cell lines as well as in keratinocytes transduced with HPV16 oncogenes when compared to normal PHK (Supplementary Figs 2 and 3). Conversely, no alterations in these proteins expression were observed in keratinocytes expressing HPV11 proteins. These results indicate that up-regulation of these proteins may be a characteristic of cells infected by high-risk HPV types. Moreover, the their consistent over expression in both HPV-positive and HPV-negative tumor cells suggests that they may be important to cell transformation process leading to cervical cancer.

When we analyzed CHEK2 levels we observed that its expression is higher in the HPV-negative C33A cell line compared to HeLa and SiHa cells. This result confirms the ones obtained after analyzing the mRNA levels (Table 2). Intriguingly, we detected that CHEK2 levels were upregulated in all transduced keratinocytes when compared to PHK, suggesting that this may be a consequence of retroviral infection (Supplementary Fig. 2). However, the biological significance of this result is, at present, unknown.

The protein MAP2K6 was detected only in HeLa and SiHa cells extracts (Supplementary Fig. 2). Since this protein was not detected in PHK acutely expressing HPV11 or HPV16 E6 and E7 proteins we hypothesize that this factor may be involved in late stages of cervical carcinogenesis. However, the effect of other HPV proteins on MAP2K6 deregulation during the course of viral infection and HPV-associated lesions progression cannot be excluded. Finally, we observed a slight increase in PNK expression only in HeLa cells and in PHK expressing HPV11 E6 and E7 when compared to PHK.

**Figure 1:**
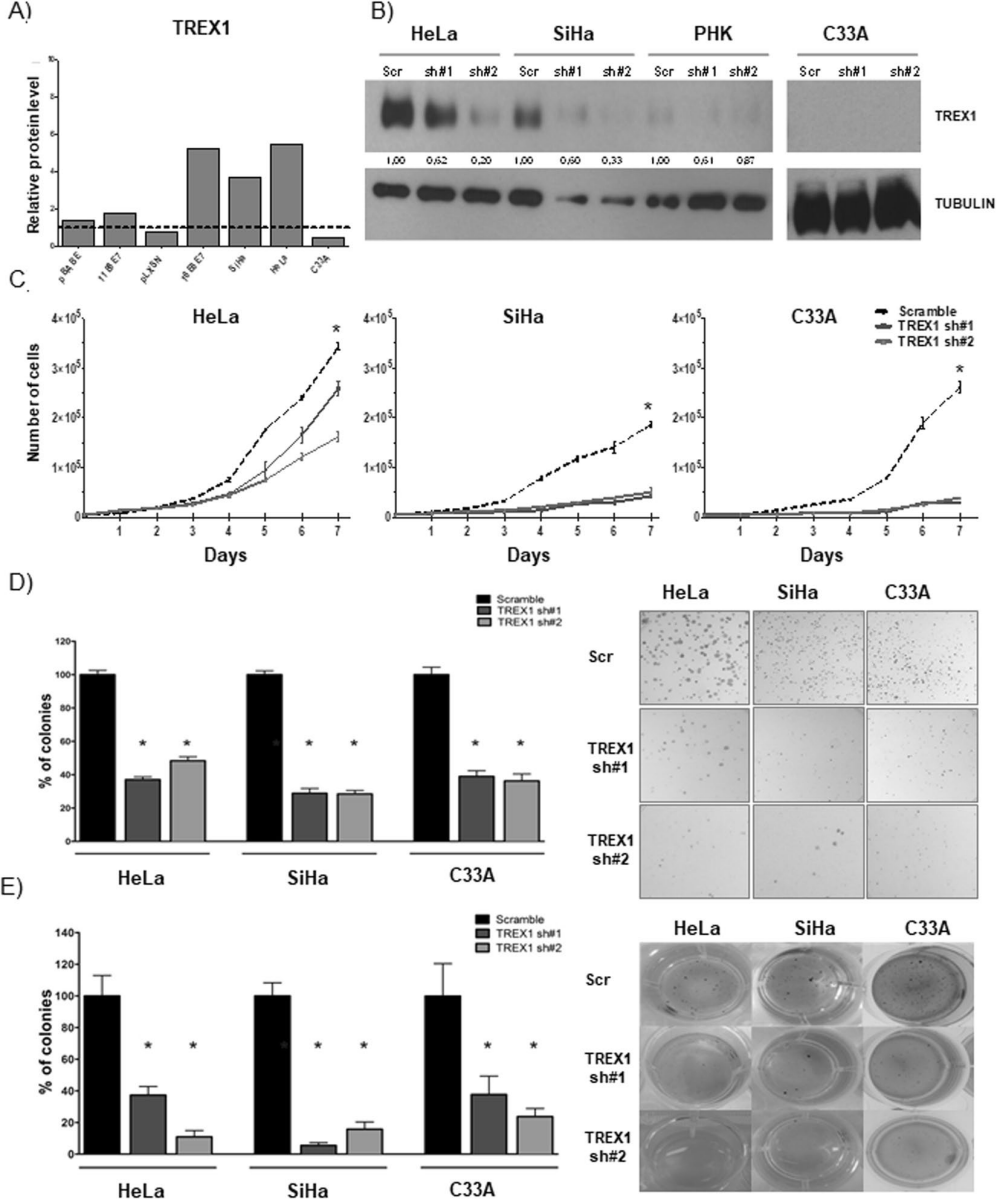
TREX1 silencing inhibits proliferation, clonogenic and anchorage independent growth of cervical cancer derived cell lines. (**A**) The levels of TREX1 were determined by western blot using 30 μg of total protein extracts from monolayer cultures of primary human keratinocytes (PHK), PHK transduced with HPV11 (PHK pBabe, PHK pBabe 11E6E7) or HPV16 (PHK pLXSN, PHK pLXSN 16E6E7) genes and cervical cancer derived cell lines C33A, SiHa (HPV16) and HeLa (HPV18). (**B**) The expression of TREX1 in C33A, SiHa and HeLa cells was silenced using lentiviral particles expressing specific shRNAs. Silencing efficiency was determined by western blot as described in A. (**C**) For proliferation assays (growth curves) 5000 cells of each cell line were seeded in 6-well plates and counted daily for seven days. (**D**) For clonogenic assays 1000 cells of each cell line were seeded in 100 mm Petri dishes and cultured for 15 days. Colonies were stained with crystal violet and counted. (**E**) For anchorage independent growth 500 cells of each cell type were seeded in 24 well plates in 0,6% agarose prepared in M10. After 30 days colonies were stained with MTT and counted. The results shown are representative of at least three independent experiments performed in triplicate. *p-value ≤ 0.05. Western blot signals were quantified using ImageJ software using housekeeping genes actin or tubulin as normalizers and presented as expression relative to normal keratinocytes.

The expression profile of the exonuclease TREX1 was unique among the proteins analyzed. TREX1 levels were upregulated exclusively in HPV-positive tumor cell lines and in PHK expressing HPV16 oncogenes (Fig. 1A). Altogether our results confirm the existence of major alterations in the expression of proteins involved in several DNA damage repair/signaling pathways in the cells under study. Most of these alterations are readily observed in tumor cells. Besides, a subset of proteins is clearly affected by the expression of HPV proteins suggesting that they may play a role in the development of HPV-associated diseases.

### TREX1 is important for cervical cancer derived cell lines viability

TREX1 was selected for further analysis because its expression at the protein level is upregulated in cells expressing HPV oncogenes namely; PHK acutely transduced with HPV16 E6 and E7 genes or HPV-positive cervical cancer derived cell lines SiHa and HeLa. On the other hand, lower or undetectable levels of this protein were observed in HPV-negative PHK and C33A cells, respectively (Tables 1, 2 and Fig. 1A).

The expression of TREX1 was silenced in C33A, HeLa and SiHa cells using lentiviral vectors expressing specific shRNAs and was confirmed by western blot analysis in whole cell protein extracts (Fig. 1B). As a silencing specificity control we used protein extracts from cultures of the same cells transduced with lentivirus expressing a shRNA scrambled sequence with no reported cellular targets.

First, we analyzed the effect of TREX1 silencing on C33A, SiHa and HeLa cells proliferation by performing growth curves. The results presented in Fig. 1C show that TREX1 down-regulation inhibits these cells growth. Furthermore, we observed that silencing of this gene greatly affects the clonogenic potential of cervical cancer derived cell lines (Fig. 1D). In view of these results we decided to test further the tumorigenic potential of cervical cancer cell lines upon TREX1 silencing. The soft agar colony formation is considered a stringent test for determining tumorigenic potential of cells. Therefore, we determined the effect of TREX1 downregulation in cervical cancer cell lines capability of forming colonies in soft agar. Our results clearly show that TREX1 is critical for anchorage-independent growth of these cell lines since its silencing reduced more than 60% the number of colonies in the three cell lines tested (Fig. 1E). Besides, the colonies observed after TREX1 silencing were smaller than the ones observed in cultures of control cells (transduced with lentivirus expressing scrambled shRNA). Altogether, our results indicate that TREX1 is important for the proliferation and maintenance of tumorigenic traits in cervical cancer derived cell lines.

### TREX1 is upregulated in cells expressing HPV oncogenes

**Figure 2:**
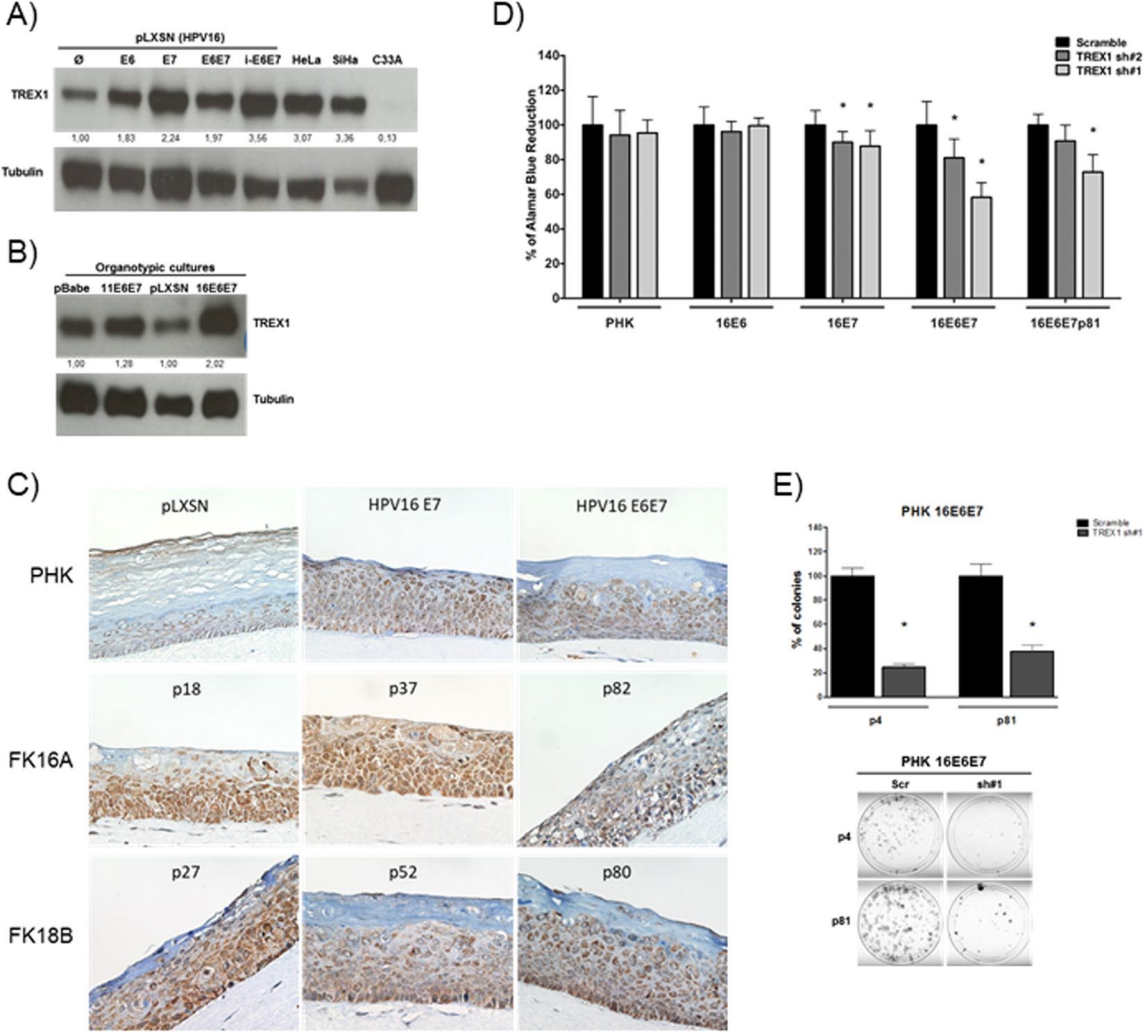
HPV16 oncogenes induce TREX1 expression in human keratinocytes and sensitize these cells to TREX1 silencing. TREX1 expression levels were determined by western blot in 30 micrograms of total protein extracts from monolayer (**A**) and organotypic (**B**) cultures of keratinocytes transduced with constructs expressing E6 and E7 from HPV16 and HPV11. (**C**) TREX1 expression was analyzed by immunohistochemistry in sections from organotypic cultures established from low passage number keratinocytes transduced with retroviral vectors expressing HPV16 E6 and/or E7 with. Besides, organotypic cultures established from keratinocytes transfected with HPV16 or HPV18 whole genomes and cultured for different passage number (p18 to p82) were used. Magnification: X400. (**D**) The effect of TREX1 silencing on cell viability was determined in control or HPV16 E6 and/or E7 transduced PHKs. Gene silencing was performed as described in Fig. 1. Cells were cultured in 96 wells plates (2000 cells/well) and after 72 hours 10 µL of Alamar blue per well were added. Cells were incubated at 37 °C and Alamar Blue’s reduction was monitored every hour in a spectrophotometer through absorbance measurement at 570 e 600 nm. (**E**) Clonogenic assays with low- (p4) and high-passage (p81) PHK expressing HPV16 E6 and E7 were performed as described in Fig. 1. The results shown are representative of at least three independent experiments performed in triplicate. *p-value ≤ 0.05.

The results described above indicate that TREX1 is upregulated in cells expressing HPV genes. In order to identify the viral genes involved in this process we analyzed TREX1 protein levels in monolayer cultures of PHK expressing HPV16 E6 and/or E7. The results presented in Fig. 2A show that cells expressing either HPV16 oncogene exhibit higher levels of TREX1 protein. This effect was confirmed in protein extracts from organotypic raft cultures established from PHK transduced with E6 and E7 from HPV11 and HPV16 (Fig. 2B). Besides, we analyzed the expression of TREX1 in rafts established from keratinocytes harboring the complete genome of HPV16 and HPV18 at different passages (p18 to p82). These cultures reproduce histological alterations characteristics of low and high-grade cervical lesions as previously reported^32^. Our results confirm the observation made in monolayer cultures that TREX1 levels are upregulated in cells expressing HPV16 E6 and/or E7 when compared to control cultures (Fig. 2B,C, and data not shown). Moreover, immunohistochemistry analysis showed that organotypic cultures of PHK harboring HPV16 or HPV18 whole genomes exhibited stronger TREX1 staining than those established from control keratinocytes (Fig. 2C). Conversely, no alterations in TREX1 levels were observed in protein extracts obtained from raft cultures established from PHK expressing the low-risk HPV11 E6 and E7 (Fig. 2B and data not shown). These results suggest that E6 and E7 from high-risk HPV types are involved in TREX1 upregulation.

### Dependence on TREX1 is associated with E7 expression

It has been well established that sustained expression of E6 and E7 proteins is required to maintain the transformed phenotype. Therefore, we investigated the role of these proteins in the sensitization of HPV-positive cells to TREX1 silencing. We observed that control and E6 expressing PHK were unaffected by TREX1 silencing. However, cells expressing HPV16 E7 exhibited reduced viability upon TREX1 silencing (Fig. 2D). Moreover, concurrent E6 and E7 expression turned PHK highly sensitive to TREX1 silencing affecting both their proliferative and clonogenic potential (Fig. 2D,E). Interestingly, the effect of TREX1 silencing was also observed in keratinocytes immortalized with HPV16 E6/E7 and cultured for 81 passages. These results show that HPV16 oncoproteins expression, mainly E7, reduces PHK viability upon TREX1 silencing.

### TREX1 silencing is associated with p53 upregulation, accumulation of subG1 cell population and downregulation of proliferation markers

**Figure 3:**
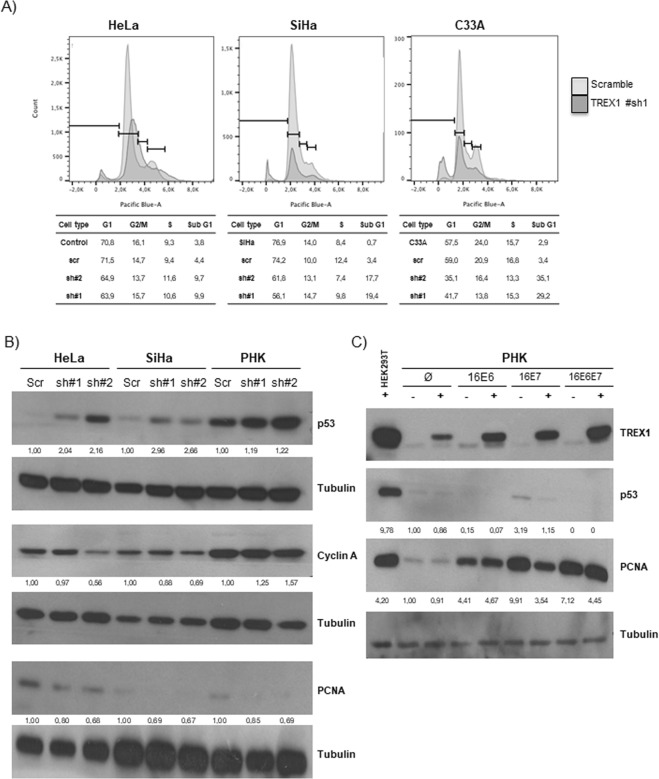
TREX1 silencing causes accumulation of sub-G1 cells and p53 upregulation. TREX1 silencing in SiHa, HeLa, C33A and PHK was performed as described in Fig. 1. (**A**) For cell cycle study, monolayers cultures of the different cell lines silenced for TREX1 expression were seeded in triplicate in 24 well plates (5000 cells/well). After 5 days cells and supernatants were harvested and analyzed in a FACSCalibur. At least 10,000 events were acquired for each condition. The data obtained were analyzed with the FlowJo software. (**B**) To analyze the effect of TREX1 silencing on the levels of regulators of the cell cycle 30 μg of total protein extracts from monolayer cultures of each cell type were analyzed using antibodies against cyclin A, PCNA, p53 and TREX1. (**C**) To determine the effect of TREX1 superexpression on the levels of regulators of the cell cycle in PHK expressing HPV16 E6 and/or E7 the cells were transfected with pcDNA5 expression vector harboring the TREX1 sequence. The analysis of the expression of PCNA, p53 and TREX1 was performed as described in (**B**). Western blot signals were quantified using ImageJ software using housekeeping genes actin or tubulin as normalizers and presented as expression relative to normal keratinocytes.

Next, we investigated the molecular mechanisms underlying the loss of cell fitness resulting from TREX1 silencing. For this purpose we used flow cytometry to analyze the cell cycle of cervical cancer cells upon gene silencing. Our results indicate that TREX1 silencing is associated with an increase in the subG1 population in the three cervical cancer cell lines analyzed (Fig. 3A) which points to cell death as the main outcome.

It has been previously reported that *Trex1*^*−/−*^ mouse embryonic fibroblasts (MEF) exhibit increased p53 levels in the absence of exogenous stimuli^33^. Therefore, we investigated the expression levels of p53 in cervical cancer derived cell lines. Our results show that HeLa and SiHa cells exhibit a clear upregulation in p53 levels upon TREX1 silencing (Fig. 3B). This effect is paralleled by the downregulation of cell proliferation markers such as PCNA and cyclin A. Interestingly, these effects are less evident in normal PHK.

Since the effect of TREX1 silencing depends on the presence of HPV oncogenes we analyzed the effect of TREX1 super expression in PHK transduced with HPV16 E6 and/or E7. The results presented in Fig. 3C show that TREX1 expression resulted in reduced p53 levels, particularly in normal PHK and PHK expressing HPV16 E7 alone. Alterations in the expression levels of p53 were marginal or not observed in cell expressing HPV16 E6, either alone or in combination with E7, probably due to the intrinsically low p53 levels exhibited by these cells.

Altogether, our results show that TREX1 silencing induces cell death, a fact that is paralleled by an increase in p53 levels and a drop in the expression of factors involved in cell proliferation.

### TREX1 is up-regulated in CIN 3 and invasive cervical carcinoma

The results described above prompted us to analyze the levels of TREX1 in the context of cervical pathology. For this purpose, TREX1 levels were determined in cervical biopsies from patients with cervicitis (n = 16), cervical intraepithelial neoplasia grade 2 (CIN2) (n = 15), CIN3 (n = 15), invasive squamous cell carcinoma (SCC) (n = 15) and adenocarcinoma (n = 15).

TREX1 expression was detected by IHC and a score was assigned to each sample according to the percentage of stained cells as follows: 0: Negative; (1): <5% of cellular positive immunoreaction; (2): 5–50% of cellular positive immunoreaction; and (3): >50% of cellular positive immunoreaction. We observed that 25% of cervicitis samples were negative for TREX1 immunostaining (not shown). Furthermore, all TREX1 positive cervicitis exhibited weak/moderate TREX1 signal (≤50% of stained cells). Conversely, the percentage of samples with strong TREX1 signal increased steadily from CIN2 (27%) to CIN3 (53%) and SCC (73%) cases. Besides, most of the adenocarcinomas samples (93.3%) exhibited >50% of cellular positive immunoreaction (Table 3).

**Table 3:** Determination of TREX1 expression in cervical samples.

	Cervicitis	CIN2	CIN3	SCC	Adeno
Cases (%)	16 (100,00)	15 (100,00)	15 (100,00)	15 (100,00)	15 (100,00)
Weak/Moderate*	16 (100,00)	11 (73,33)	7 (46,67)	4 (26,67)	1 (6,67)
Strong**	0 (0,00)	4 (27,67)	8 (53,33)	11 (73,33)	14 (93,33)

n = 76; *Weak/moderate = no staining or ≤50% of stained cells; **Strong = > 50% of stained cells.

**Figure 4:**
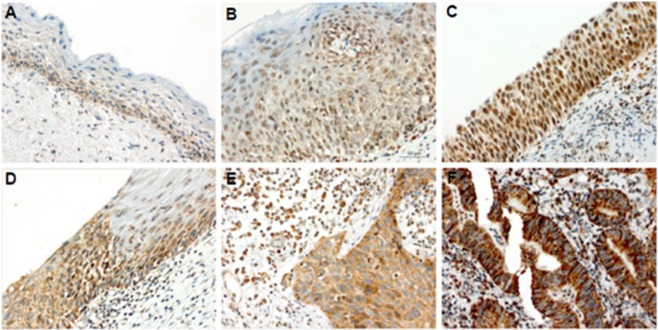
TREX1 is upregulated in CIN 2/3 and invasive carcinoma samples. A representative immunoreactivity of TREX1 in clinical samples is shown. (**A**) A sample of cervicitis showing a positive reaction for TREX1 predominantly decorating the basal layer. (**B**) A cervical intraepithelial neoplasia grade 2 (CIN2) sample exhibiting a positive reaction weakly staining the basal and parabasal layers of stratified epithelium. (**C**) A CIN3 (or *in situ* carcinoma) sample strongly stained by TREX1 immunoreaction, mainly expressed at nuclei. (**D**) Highlight of the junction between a normal epithelium (right side) and a CIN3 lesion (left side). (**E**) Example of an invasive squamous cells carcinoma with cytoplasmic positive reaction for TREX1. (**F**) Example of an intense positive reaction for TREX1 decorating the cytoplasmic area of an invasive cervical adenocarcinoma. Note the strong TREX1 cytoplasmic staining in CIN3 and carcinoma with sparse nuclear staining (**D**–**F**). Magnification: X400.

Figure 4 shows a representative immunostaining for TREX1 in cervical samples. In cervicitis tissue, weak/moderate TREX1 signal was observed in the cytoplasm and nuclei of cells in the lower layers of cervical epithelium (Fig. 4A and right side of 4D). On the other hand, CIN2/3 and invasive carcinoma samples displayed mainly strong cytoplasmic TREX1 staining throughout the lesion (Fig. 4B–F).

Our observations in cervical samples show that TREX1 upregulation may correlate with lesion progression during cervical disease. Taken together, our results suggest that TREX1 may play a role during cervical cancer onset/progression.

### TREX1 expression is upregulated in CIN and invasive cervical clinical samples and it negatively correlates with miR103 levels in CIN3 lesions

**Figure 5:**
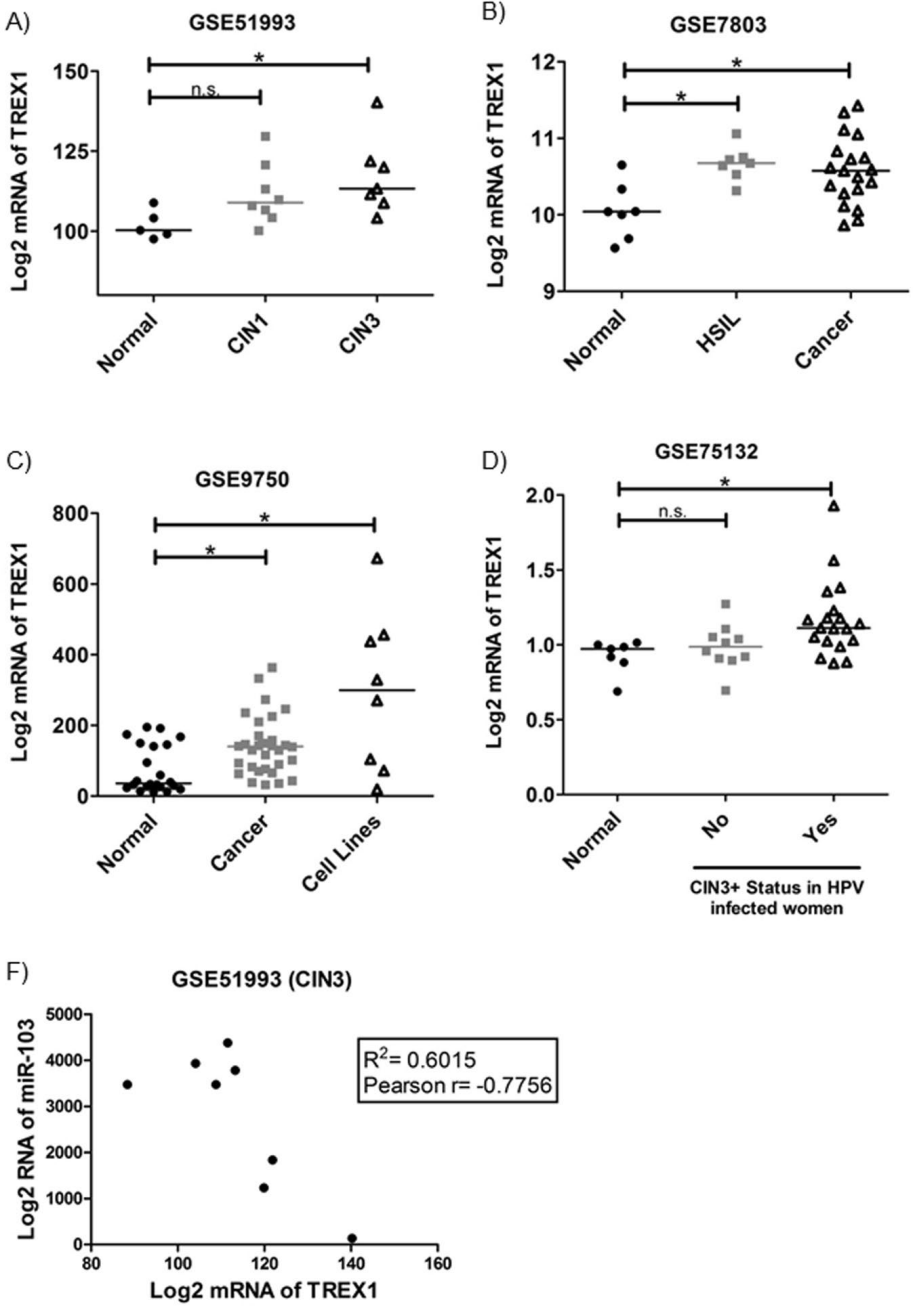
TREX1 mRNA levels are up-regulated in CIN and invasive carcinomas. TREX1 mRNA levels were analyzed in CIN and SCC series form GEO expression datasets. (**A**) The mRNA expression of TREX1 was upregulated in CIN1 and CIN3 when compared with normal cervical tissues in GSE51993 series. (**B**,**C**) The mRNA expression of TREX1 was increased in invasive carcinomas and cervical cancer cell lines when compared with normal cervical tissues in two different series GSE7803 and GSE9750. Note the TREX1 mRNA is also upregulated in high-grade squamous intraepithelial lesions (HSIL). (**D**) The mRNA expression of TREX1 was upregulated in HPV-infected women who developed CIN3+ lesions when compared with HPV- infected women without lesions or HPV-negative women (GSE75132). (**E**) The levels of TREX1 mRNA exhibit a moderate negative correlation with miR103 expression in CIN3 (GSE51993).

To further address the possible role of TREX1 expression in the natural history of cervical cancer, we explored four cervical cancer expressions array series from Gene Expression Omnibus (GEO) dataset (https://www.ncbi.nlm.nih.gov/gds). The *in silico* analysis of the datasets showed that TREX1 mRNA expression was significantly higher in CIN1 (n = 8) and CIN3 (n = 7) lesions than in normal adjacent tissues of early lesions (n = 5) (Fig. 5A; p = 0.0234). TREX1 mRNA level was also increased in High-Grade Squamous Intraepithelial Lesion (HSIL) (n = 7) and cervical tumor tissues (n = 19) when compared to normal cervical hysterectomy specimens resected for benign disease using laser capture micro dissection (n = 7)^34^ (Fig. 5B; p = 0.0140). In another dataset from GEO (GSE9750), we observed that TREX1 mRNA expression was up regulated in invasive carcinomas (n = 33) and cervical cancer cell lines (n = 8) when compared with age-matched normal cervical tissues from hysterectomy specimens (n = 19)^35^ (Fig. 5C; p = 0.0019). Furthermore, TREX1 mRNA level was increased in women persistently infected with HPV who developed CIN3+ lesion (n = 19) when compared with HPV infected women that presented no lesions (n = 10) or women with no HPV infection or cervical pre-cancerous lesions (n = 7)^36^ (Fig. 5D; p = 0.0074).

Wilson and co-workers have recently shown that miR-103 negative regulation of its target gene TREX1 reprograms the tumor microenvironment^37^. Therefore, we analyzed the existence of an association between mRNA expression of TREX1 and miR103 levels in CIN1, CIN3 and normal adjacent tissue of early lesions in the GSE51993 series. Our data shows that TREX1 mRNA level presents a negative correlation with miR103 expression in CIN3 (p = 0.0237) (Fig. 5F), but not in CIN1 (p = 0.1482) or in normal tissue (p = 0.4928) (data not shown).

## Discussion

Alterations of genes involved in DNA damage repair/signaling or in their expression patterns may favor to accumulation of genetic alterations important in cancer development. In fact, genome instability is considered one defining hallmark for most cancers^38^.

In this study, we analyzed the expression profile of 135 genes involved in DNA damage repair pathways in cervical cancer derived cell lines. Our results clearly show the existence of major alterations in the expression of these genes in tumor cell lines when compared to normal keratinocytes. Interestingly, we identified 18 genes differentially expressed between the three tumor-derived cell lines analyzed and normal keratinocytes (Table 1). In addition, we identified 9 genes that distinguished HPV-positive from the HPV-negative cells (Table 2). These observations suggest that alterations in these genes expression may play a role in the natural history of cervical cancer. Moreover, deregulation of gene expression between HPV-positive and HPV-negative cells may be related to the action of specific viral factors. Further studies are required to determine the effect of HPV proteins on the expression of these genes.

The analysis of the expression pattern of the genes under study in tumor-derived cell lines produced some unexpected results. For instance, we detected a relatively small number of differentially expressed genes between HeLa cells and normal PHK. On the other hand, SiHa and C33A cells exhibited a great difference in the pattern of expression of DNA damage repair associated genes compared to normal cells. Interestingly, many of these genes were simultaneously deregulated in both SiHa and C33A cells. Consistent with this observation we identified just 3 genes, namely *CCNO, PNK* and *TREX1* that concurrently distinguished HPV-positive cell lines from normal PHK. Besides, we also observed that *MSH4, NBN, NEIL2, OGG1* and *XRCC2* were downregulated in both HPV-positive tumor cell lines when compared to the HPV-negative one. A recent study analyzed the expression of *XRCC1, ERCC2, ERCC4* and *ERCC1* in cervical tissue samples and showed that the levels of both mRNA and protein were lower in squamous intraepithelial lesions and cervical cancer samples than in samples from control patients^39^. Altogether, these observations indicate that neither HPV positivity nor the HPV type present is associated with specific alterations of the pathways analyzed here. This may reflect the existence of differences in the mechanisms of cell transformation by different HPV types, the existence of differences that are inherent to the process of tumor evolution or both.

The results discussed above highlight the presence of important differences between HPV-transformed cells. Similar results have been described before. For instance, a study that addressed the effect of kinases inhibition on cellular viability reported that the enzymes required for HeLa cells survival were dramatically different from kinases required by SiHa cells. Of note, the group of kinases important for the viability of HPV16-positive CasKi cells was strikingly similar to the one required by HeLa cells^40^.

Among the differentially expressed genes *TREX1* was selected for functional analysis. We show that the expression of TREX1, the major cytoplasmic 3′-5′ exonuclease in mammalian cells, is upregulated in cervical cancer derived cell lines. TREX1 is essential for DNA damage repair and plays an important role in the elimination of DNA present in the cytoplasm preventing the activation of innate immune mechanisms. Deficiencies in this exonuclease are associated with autoimmune diseases Aicardi-Goutières syndrome and familial chilblain lupus^41,42^. This protein forms a homodimer that locates in the cytoplasm as part of the SET complex together with nucleases APE1 and NM23-H1. This complex translocates to the nucleus during S phase or in response to genotoxic stress. Besides, it participates in granzyme A-mediated cell death to degrade nicked genomic DNA^43^. In our experimental approach we observed that TREX1 silencing greatly reduced the oncogenic potential of cervical cancer derived cell lines by dampening clonogenic and anchorage independent growth capability.

Although the molecular mechanisms underlying the inhibitory effect of TREX1 silencing on cervical cancer cell lines growth are still unknown we provide some important observations. First, we demonstrate that TREX1 is upregulated in monolayer and organotypic cultures of keratinocytes expressing HPV16 E6 and/or E7. In addition, our results indicate that this is a property of high-risk HPV types since similar observations were made in rafts expressing HPV18 oncogenes. Conversely, no alterations in TREX1 expression were detected in rafts expressing E6/E7 from the low-risk HPV11. Second, we show that HPV16 E7 oncogene expression is sufficient to reduce cell proliferation/viability upon TREX1 silencing. Third, we provide data that TREX1 silencing correlates with higher p53 levels in cervical cancer derived cell lines. This observation is paralleled by the downregulation of PCNA and cyclin A, and an increase in the proportion of subG1 cells which is suggestive of cell death by apoptosis. It has been previously shown that *TREX1*^−/−^ MEF exhibit increased p53 levels, resulting in p21^waf1/cip1^ upregulation and chronic activation of ATM-mediated DNA damage checkpoint^33^. Studies to determine the molecular mechanisms linking TREX1 silencing with p53 upregulation in the context of HPV infection are currently in progress.

The role of TREX1 in cervical cancer biology is still unknown. However, the data discussed above and several other studies underscore the role of this protein in the immune response. For instance, *TREX1*^−/−^ mice present reduced survival associated with inflammatory myocarditis^44^. Recently, Yan and coworkers^45^ observed that TREX1 inhibits type I interferon response against HIV by impeding cytosolic viral DNA accumulation in rat CD4+ lymphocytes. The authors showed that this effect depends on the action of the protein HMGB2 that associates with TREX1 in the cytosolic SET complex. Moreover, it has been shown that TREX1 negatively regulates antiviral genes activated by interferon independent pathways both in human and mouse cells^46^. In this work, it was observed that activation of antiviral genes in *TREX1*^−/−^ cells requires the adaptor STING, the kinase TBK1 and IRF3 and IRF7 transcription factors. Alterations in type I interferon regulated pathways in HPV infected cells have been previously reported^14,46,47^. Besides, HPV16 E6 mediated inhibition of IRF3 transcriptional activity and reduction in TBK1 levels in cells expressing HPV16 E6 and E7 were also observed^48^, and our unpublished data]. Altogether, our data suggest that TREX1 upregulation is important for cervical cancer cells survival probably by preventing the activation of innate antiviral mechanisms. Experiments to determine the accuracy of this hypothesis are being conducted in our laboratory.

Finally, we analyzed human cervical specimens and information from expression data series and showed that TREX1 expression steadily increases from cervicitis samples to CIN2, CIN3 and invasive squamous carcinoma samples. We also showed that this factor’s expression is very high in adenocarcinomas samples. Our observations suggest that TREX1 upregulation may correlate with lesion grade. Further studies are warranted to determine the value of TREX1 as a prognostic marker for cervical lesions. Taken together, our results indicate that TREX1 upregulation may play a role during cervical cancer onset/progression.

To our knowledge, this is the first study to address the presence of global alterations in the expression pattern of genes involved in DNA damage repair in cervical cancer cell lines. Using this approach we identified set of genes which expression patterns differentiate normal from cervical cancer derived cells. Furthermore, we provide the first evidence that TREX1 is essential for cervical tumor cells survival. We also show that E7 from high-risk HPV types and p53 downregulation play a role in this effect. Finally, we demonstrate that TREX1 levels are upregulated in high-grade cervical lesions and invasive carcinomas of the cervix. Altogether, our results suggest that TREX1 upregulation may be important in the natural history of cervical cancer. A systematic analysis to determine the existence of a correlation between TREX1 levels, lesion grade and HPV presence in a larger set of cervical samples is currently in progress. The study of the value of this protein as a biomarker of cervical disease and of its use as potential target for therapy warrant further studies.

## Materials and Methods

### Cells and retroviruses

Cervical cancer derived cell lines SiHa (HPV16), HeLa (HPV18) and C33A (HPV-negative), and human embryonic kidney cell HEK-293T were cultured in MEM (Invitrogen, Carlsbad, CA, USA) supplemented with 10% BCS (Cultilab, Campinas, SP, Brazil) and maintained at 37 °C and 5% CO_2_. Pooled neonatal foreskin keratinocytes (Lonza, Charleston, USA) were grown in serum-free medium (Invitrogen, Frederick, MD, USA). At passage one, keratinocytes were acutely infected with recombinant pLXSN-based retroviral retroviruses expressing HPV16 oncogenes and the neomycin selectable marker. These cells were used to seed organotypic cultures as described elsewhere^32,47^. Briefly, cells (2 × 10^5^ cells/well) were seeded on top dermal equivalents prepared in 24 well/plates and composed of rat-tail collagen type I and fibroblasts. After 9 days at the medium–air interface organotypic cultures were harvested by fixation in 10% buffered formalin, embedded in paraffin, and then cut into 4-µm sections. Recombinant pLXSN and pBabe vectors were a gift from Denise Galloway (Fred Hutchinson Cancer Research Center, Seattle, WA) and Dennis J. McCance (University of New Mexico, Albuquerque, NM), respectively.

### Analysis of gene expression

Total RNA was isolated using TRIzol (Invitrogen, Carlsbad, CA, USA) according to the manufacturer’s instructions. RNA samples were treated with RQ1 RNase free DNase (M198A, Promega, Madison, WI, USA) and cDNA was synthetized using the RT^2^ First Strand Kit (SABiosciences, Frederick, MD, USA) according to the manufacturer’s instructions. Finally, 1 µg of cDNA from each sample was analyzed using the DNA Damage Signaling Pathway (PAHS-029A) and The Human DNA Repair (PAHS-042A) RT² Profiler PCR Array platform (PAHS-063, SABiosciences, Frederick, MD, USA) following the manufacturer’s instructions in a ABI 7500 equipment (Applied Biosystems, Warrington, UK).

### Protein extraction and immunoblotting

Total protein extracts were obtained by incubating subconfluent cell cultures with 500 µl of cold lysis buffer (150 mM NaCl, 50 mM Tris-HCl [pH 7,5], 0,5% NP-40) with protease inhibitors (Complete, Roche Diagnostics, GR). Extracts were cleared from debris by centrifugation (10,000 × g for 20 min) at 4 °C. Supernatants protein concentrations were determined using the Bio-Rad protein assay (Bio-Rad Laboratories, Hercules, CA). Thirty μg of proteins were resolved by electrophoresis through sodium dodecyl sulfate (10 to 12%) polyacrylamide gels and transferred to polyvinylidene difluoride membranes (Amersham Pharmacia Biotech, UK). The membranes were blocked for 1 h in 5% nonfat milk, probed with primary antibodies against CHK2 (#3440, Cell Signaling, MA, USA); DNA Ligase I (ab615), FEN1 (ab17993), MEK6 (ab52937), OGG1 (ab81624), PNK (ab151418), Rad9 (ab70810), RPA70 (ab79398), SMC1 (ab21583) and TREX1 (ab185228) from Abcam (Cambridge, MA, UK); MRE11 [H-300] (sc-22767, Santa Cruz Biotechnology); PCNA (13–3900) from Zymed (Thermo-Fisher Scientific, MA,USA); actin (sc-1615), Rad51 (sc-8349) and tubulin (sc-25259) from Santa Cruz Biotechnology (CA, USA), according to the manufacturer’s instructions. Membranes were reprobed with horseradish peroxidase (HRP)-conjugated secondary antibodies and revealed using Enhanced Chemiluminescence procedures according to the manufacturer’s recommendations (Amersham Pharmacia Biotech, UK).

### Gene Silencing

shRNAs clones for TREX1 silencing selected the from the MISSION shRNA Human Gene Family Set-DNA Repair Pathway library (SH1811, Sigma-Aldrich, St. Louis, MO, USA) were transfected in HEK-293T cells together with the MISSION Lentiviral Packaging Mix (Sigma-Aldrich, St. Louis, MO, USA) using FuGENE HD Transfection Reagent (Promega, Madison, WI, USA) according to the manufacturer’s instructions. Supernatants containing lentivirus were collected after 48 and 72 hours after transfection. Lentiviral particles titer was determined using a HIV-1 p24 ELISA kit (ZeptoMetrix Corporation, Buffalo, NY, USA). Sub-confluent cultures of HeLa, SiHa and C33A cells were infected with lentiviral particles (MOI 5) expressing specific shRNA. After two days cells were selected with 2,5 µg/ml of puromycin (Sigma-Aldrich, St. Louis, MO, USA) until complete death of control cells.

### TREX1 super expression

The TREX1 sequence cloned in the pcDNA5 (Invitrogen, Carlsbad, CA, USA) was kindly provided by Dr. Chuan-Jen Wang (Department of Pharmacology, Yale University School of Medicine, New Haven, USA)^49^. The plasmids were transfected as described above.

### Cell proliferation, clonogenic and anchorage independent growth analysis

For proliferation analysis cells transduced with the shRNA described above seeded in octuplicate in 96 wells plates (2000 cells/well). After 72 hours 10 µL of Alamar blue (Invitrogen, Carlsbad, CA, USA) were added per well and cells were incubated at 37 °C for 4 to 7 hours. After this period, Alamar Blue reduction was monitored through absorbance measurement at 570 e 600 nm in an Epoch Microplate Spectrophotometer (Bio-Tek, VT, USA). For clonogenic analysis one-thousand SiHa, HeLa and C33A cells infected and selected as described above were seeded in triplicate in 100 mm plates. After 15 days cultures were stained with crystal violet and colonies were counted. For anchorage independent growth analysis five-hundred cells of each type were suspended in triplicate in 500 µl of M10 with 0,6% agarose and seeded in a 24 wells plate on top 1 ml of 1% agarose in M10. After 30 days colonies were stained with (3-(4,5-dimethylthiazolyl-2)-2,5-diphenyltetrazolium bromide (MTT) and counted. In all cases, the results shown are representative of three independent experiments.

### Cell cycle analysis

Cells silenced for TREX1 expression were seeded in triplicate in 24 well plates (5000 cells/well). After 5 days cells and supernatants were harvested and centrifuged at 1500 rpm for 5 minutes. Cells were ressuspended in1 mL of 70% ethanol and stored at 4 °C. For cell cycle analysis cells were washed 1X with PBS and incubated with 500 μL of a solution of DAPI (4′,6-diamidino-2-phenylindole) for 30 min. Flow cytometry was performed in a FACSCalibur (BD Biosciences, NJ, USA), where at least 10,000 events were acquired. Data obtained were analyzed with FlowJo software (FlowJo Enterprise, OR, EUA).

### Tissue samples and immunohistochemistry

Cervical tissue samples were obtained from women managed in the Women’s Hospital, State University of Campinas, Brazil, between 2005 and 2011. All participants signed informed consent for sample collection. Ethical approval for this study was granted by the Comitê de Ética em Pesquisa of State University of Campinas (#1.647.260). Samples were selected retrospectively, in a completely anonymous way. All samples were collected at least three years before the study. Seventy-six paraffin-embedded cervical tissue specimens including 16 cervicitis, 15 cervical intraepithelial neoplasia grade 2 (CIN2), 15 CIN3, 15 invasive squamous carcinomas and 15 adenocarcinomas were included in the study. All methods were performed in accordance with present guidelines and regulations. Immunohistochemistry was performed using an anti-TREX1 (ab83890, Abcam, Cambridge, MA, UK) antibody at 1:150 dilution in a Ventana Benchmark GX equipment (Ventana Medical Systems, AZ, USA) according to the manufacturer’s recommended protocol. Samples were then analyzed by ALF. The positive immunoreactions were scored semi-quantitatively as: 0: Negative; (1): <5% of cellular positive immunoreaction; (2): 5–50% of cellular positive immunoreaction; and (3): >50% of cellular positive immunoreaction. For analysis proposes, the reactions were grouped in <3 (negative/weak/moderate) and = 3 (strong) categories.

### Expression datasets

GSE7803, GSE9750, GSE51993 and GSE75132 cervical cancer datasets were downloaded from the Gene Expression Omnibus (GEO http://www.ncbi.nlm.nih.gov/geo). Statistical analyses were performed using GraphPad Prism version 5.00 for Windows (GraphPad Software, La Jolla, CA, USA). One-way ANOVA analysis with Kruskal-Wallis test was applied to compare groups. Correlation of the expression of specific genes was evaluated using Pearson test. All statistical tests were two-sided. All datasets were analyzed for outliers using https://graphpad.com/quickcalcs/grubbs1/. P-value less than 0.05 was considered statistically significant.

## Supplementary information

Supplementary Figures 1-2-3

Complete blots

## Data Availability

The datasets generated during and/or analyzed during the current study are available from the corresponding author on reasonable request. All data generated or analyzed during this study, including appropriate references, are included in this article (and its Supplementary Information files).
