# Supplementary material for: Three Prime Repair Exonuclease 1 (TREX1) expression correlates with cervical cancer cells growth in vitro and disease progression in vivo
Source: Sci Rep. 2019 Jan 23;9:351. doi: 10.1038/s41598-018-37064-x (PMC6344518; doi:10.1038/s41598-018-37064-x)
Supplement: Supplementary file 1 — Supplementary Figures 1-2-3 [file 41598_2018_37064_MOESM1_ESM.pdf]

Three Prime Repair Exonuclease 1 (TREX1) expression correlates with cervical cancer cells growth *in vitro* and disease progression *in vivo*.

Bruna Prati[1], Walason da Silva Abjaude[1], Lara Termini[2], Mirian Morale[3], Suellen Herbster[1], Adhemar Longatto-Filho[4,5,6], Rafaella Almeida Lima Nunes [2], Lizeth Carolina Córdoba Camacho[1&], Silvia Helena Rabelo-Santos[7], Luiz Carlos Zeferino[8], Francisco Aguayo[9,10], Enrique Boccardo[1]*.

[1]Department of Microbiology, Institute of Biomedical Sciences, University of São Paulo (USP), Av. Prof. Lineu Prestes 1374, 05508-900, São Paulo, SP, Brazil.

[2]Centro de Investigação Translacional em Oncologia (LIM24), Instituto do Câncer do Estado de São Paulo (ICESP), São Paulo, Brazil.

[3]Department of Biochemistry, Institute of Chemistry, USP, São Paulo, Brazil

[4]Laboratory of Medical Investigation (LIM 14), Department of Pathology, School of Medicine, USP, Av. Dr. Arnaldo 455, São Paulo, 01246-903, Brazil

[5]Life and Health Sciences Research Institute, School of Health Sciences, ICVS/3B's - PT Government Associate Laboratory, University of Minho, Braga, Guimarães, Portugal

[6]Molecular Oncology Research Center, Barretos Cancer Hospital, Pio XII Foundation, Barretos, Rua Antenor Duarte Villela, 1331, Barretos, 14784-400, Brazil

[7]School of Pharmacy, Federal University of Goiás, Avenida Universitária, 74605-220 Goiânia, GO, Brazil.

[8]School of Medical Sciences, State University of Campinas (UNICAMP), Rua Alexander Fleming 101, 13083-881 Campinas, SP, Brazil.

[9]Basic and Clinical Oncology Department, Faculty of Medicine, University of Chile, Santiago, Chile.

[10]Advanced Center for Chronic Diseases (ACCDiS), Pontificia Universidad Católica de Chile, Santiago, Chile

[&]Present Address: Laboratório de Oncologia Experimental, Departamento de Radiologia, Faculdade de Medicina, USP, São Paulo, SP, Brazil. Centro de Investigação Translacional em Oncologia, ICESP, São Paulo, SP, Brazil

***Corresponding Author:** Enrique Boccardo. Department of Microbiology, Institute of Biomedical Science II, University of São Paulo. Av. Lineu Prestes, 1374- Room 239 - Cidade Universitária - São Paulo (SP) - Brazil - Phone: 55-11 3091-7292.

E-mail: eboccardo@usp.br

Supplementary figure 1 Prati *et al.*

A)

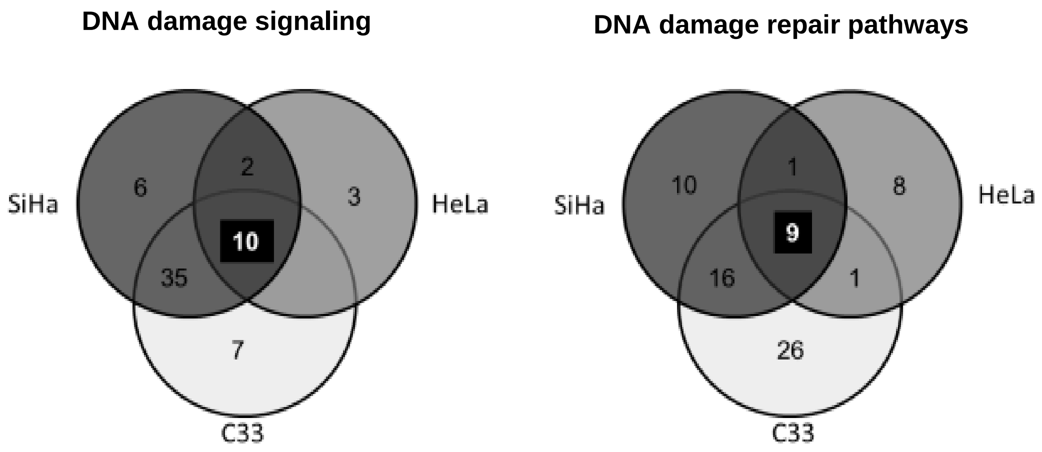

B)

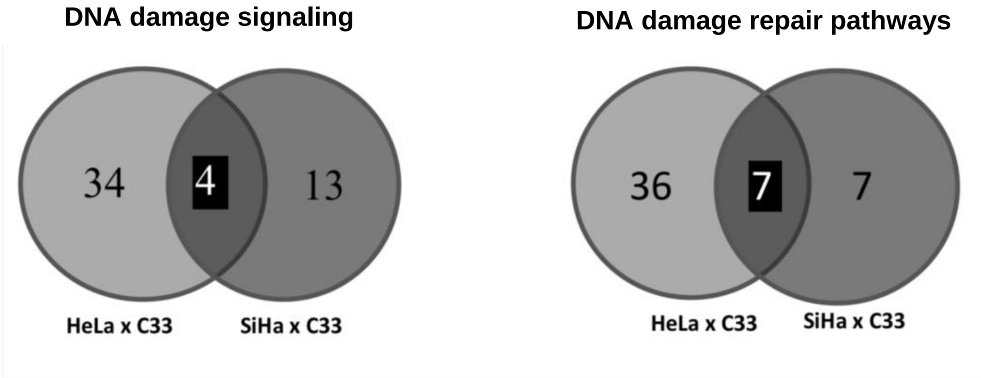

Supplementary figure 1 - In order to identify alterations in DNA damage repair pathways in cervical cancer derived cell lines we analyzed t using commercial RT-PCR arrays. The expression of 135 genes involved in DNA damage signaling and repair was analyzed in total RNA samples from SiHa (HPV16), HeLa (HPV18) and C33A (HPV negative) cell lines were analyzed using primary human keratinocytes (PHK) as the reference group (A). In a second approach the expression of the genes described above was compared between HPV-positive cells and C33A (B).

Supplementary figure 2 Prati *et al.*

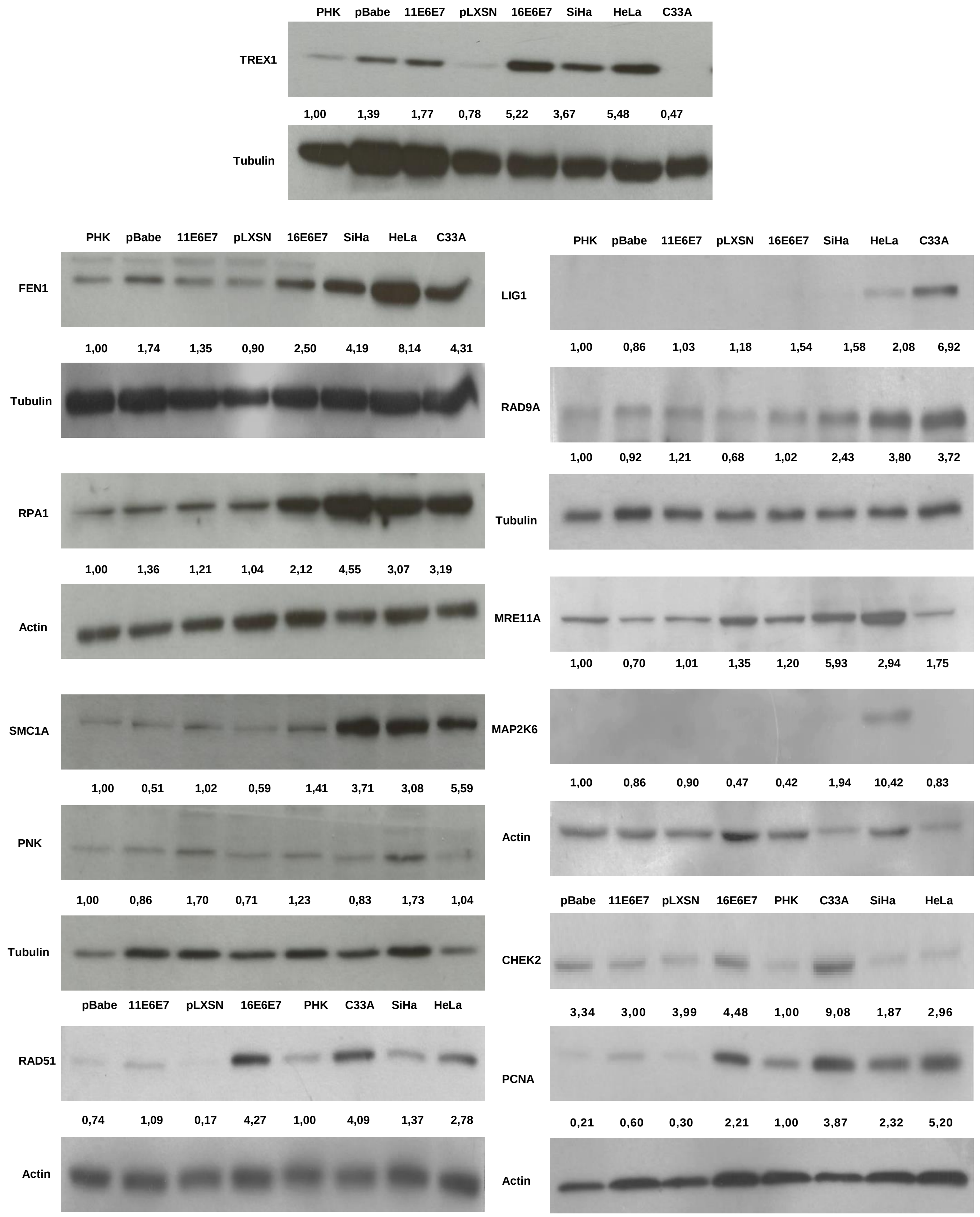

Supplementary figure 2 - The levels of proteins coded by genes differentially expressed between cervical cancer derived cell lines and PHKs were determined by western blot. Thirty µg of total protein extracts from monolayer cultures of primary human keratinocytes (PHK), PHK transduced with HPV11 (PHK pBabe, PHK pBabe 11E6E7) or HPV16 (PHK pLXSN, PHK pLXSN 16E6E7) genes and cervical cancer derived cell lines C33A, SiHa (HPV16) e HeLa (HPV18) were analyzed using antibodies against CHEK2, FEN1, LIG1, MAP2K6, MRE11A, PCNA, PNK, RAD51, RAD9, RPA1 and SMC1A. Signals obtained were quantified using ImageJ software using housekeeping genes actin or tubulin as normalizers and presented as expression relative to normal keratinocytes.

Supplementary figure 3 Prati *et al.*

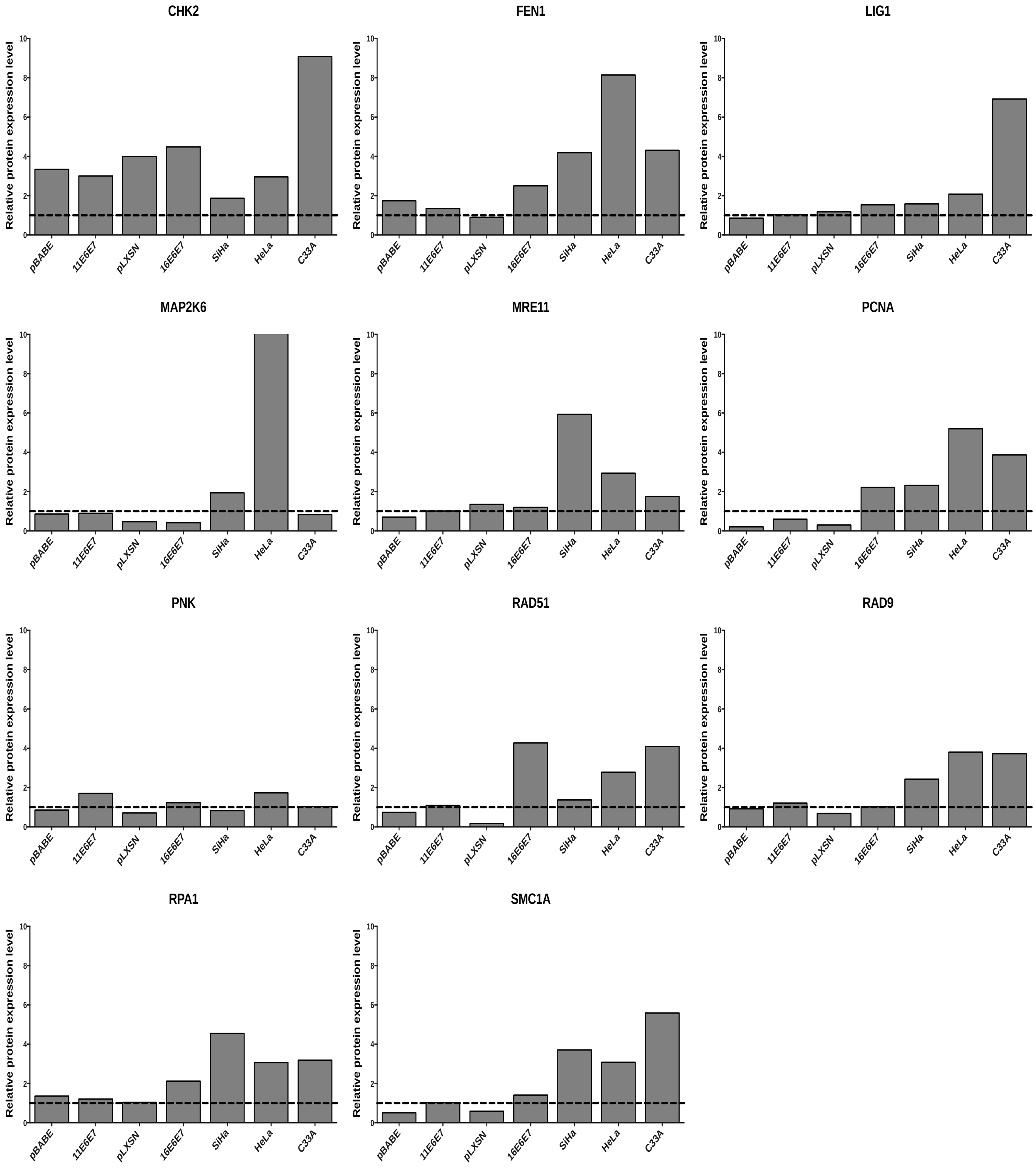

Supplementary figure 3 – Quantification of the blots described in Supplementary figure 2. Western blot signals were quantified using ImageJ software using housekeeping genes actin or tubulin as normalizers and presented as expression relative to normal keratinocytes.
