# Supplementary material for: Three Prime Repair Exonuclease 1 (TREX1) expression correlates with cervical cancer cells growth in vitro and disease progression in vivo
Source: Sci Rep. 2019 Jan 23;9:351. doi: 10.1038/s41598-018-37064-x (PMC6344518; doi:10.1038/s41598-018-37064-x)

Three Prime Repair Exonuclease 1 (TREX1) expression correlates with cervical cancer cells growth *in vitro* and disease progression *in vivo*.

Bruna Prati[1], Walason da Silva Abjaude[1], Lara Termini[2], Mirian Morale[3], Suellen Herbster[1], Adhemar Longatto-Filho[4,5,6], Rafaella Almeida Lima Nunes [2], Lizeth Carolina Córdoba Camacho[1&], Silvia Helena Rabelo-Santos[7], Luiz Carlos Zeferino[8], Francisco Aguayo[9,10], Enrique Boccardo[1]*.

[1]Department of Microbiology, Institute of Biomedical Sciences, University of São Paulo (USP), Av. Prof. Lineu Prestes 1374, 05508-900, São Paulo, SP, Brazil.

[2]Centro de Investigação Translacional em Oncologia (LIM24), Instituto do Câncer do Estado de São Paulo (ICESP), São Paulo, Brazil.

[3]Department of Biochemistry, Institute of Chemistry, USP, São Paulo, Brazil

[4]Laboratory of Medical Investigation (LIM 14), Department of Pathology, School of Medicine, USP, Av. Dr. Arnaldo 455, São Paulo, 01246-903, Brazil

[5]Life and Health Sciences Research Institute, School of Health Sciences, ICVS/3B's - PT Government Associate Laboratory, University of Minho, Braga, Guimarães, Portugal

[6]Molecular Oncology Research Center, Barretos Cancer Hospital, Pio XII Foundation, Barretos, Rua Antenor Duarte Villela, 1331, Barretos, 14784-400, Brazil

[7]School of Pharmacy, Federal University of Goiás, Avenida Universitária, 74605-220 Goiânia, GO, Brazil.

[8]School of Medical Sciences, State University of Campinas (UNICAMP), Rua Alexander Fleming 101, 13083-881 Campinas, SP, Brazil.

[9]Basic and Clinical Oncology Department, Faculty of Medicine, University of Chile, Santiago, Chile.

[10]Advanced Center for Chronic Diseases (ACCDiS), Pontificia Universidad Católica de Chile, Santiago, Chile

[&]Present Address: Laboratório de Oncologia Experimental, Departamento de Radiologia, Faculdade de Medicina, USP, São Paulo, SP, Brazil. Centro de Investigação Translacional em Oncologia, ICESP, São Paulo, SP, Brazil

***Corresponding Author:** Enrique Boccardo. Department of Microbiology, Institute of Biomedical Science II, University of São Paulo. Av. Lineu Prestes, 1374- Room 239 - Cidade Universitária - São Paulo (SP) - Brazil - Phone: 55-11 3091-7292.

E-mail: eboccardo@usp.br

Prati et al. blots for Figure 1

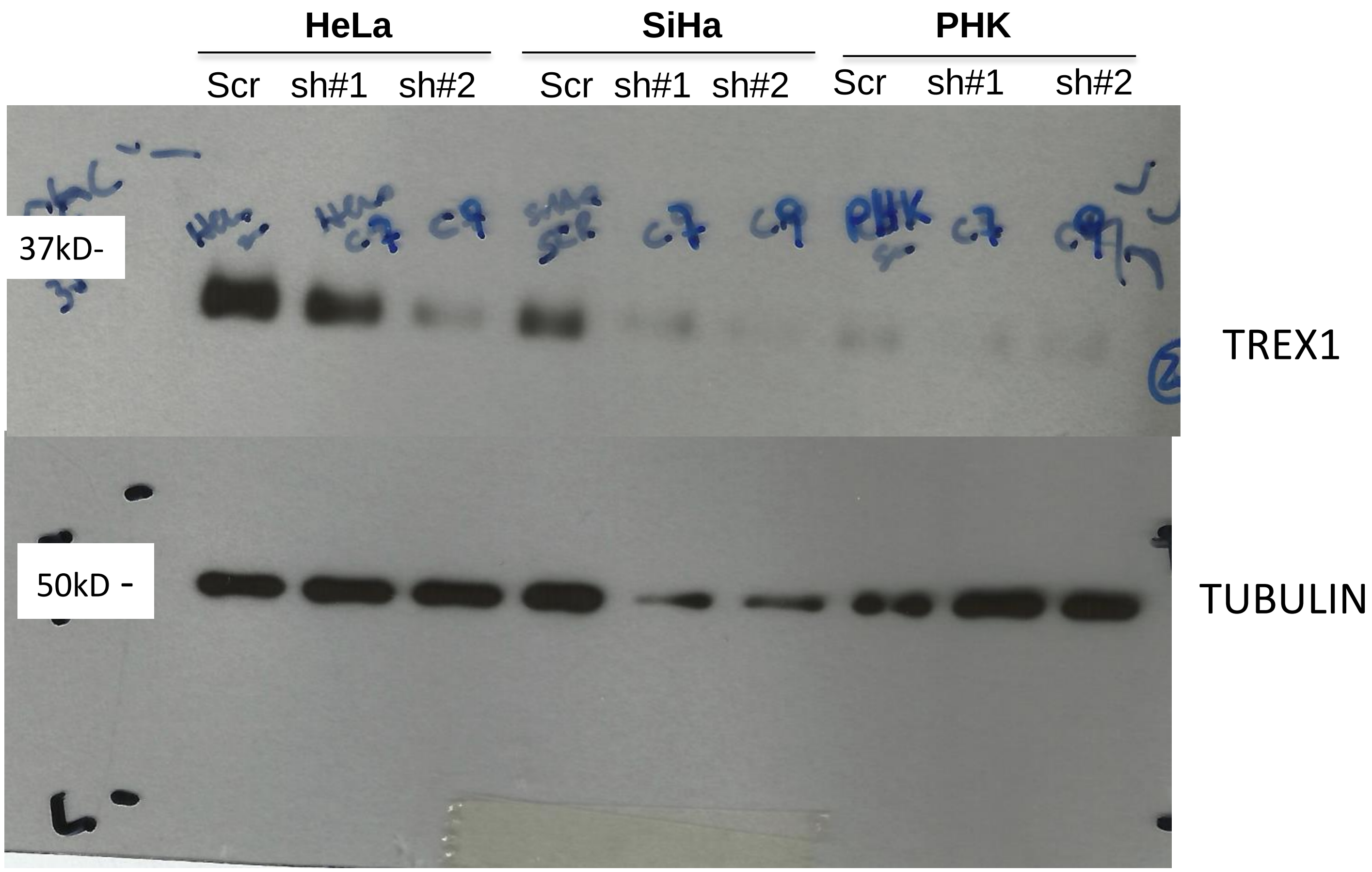

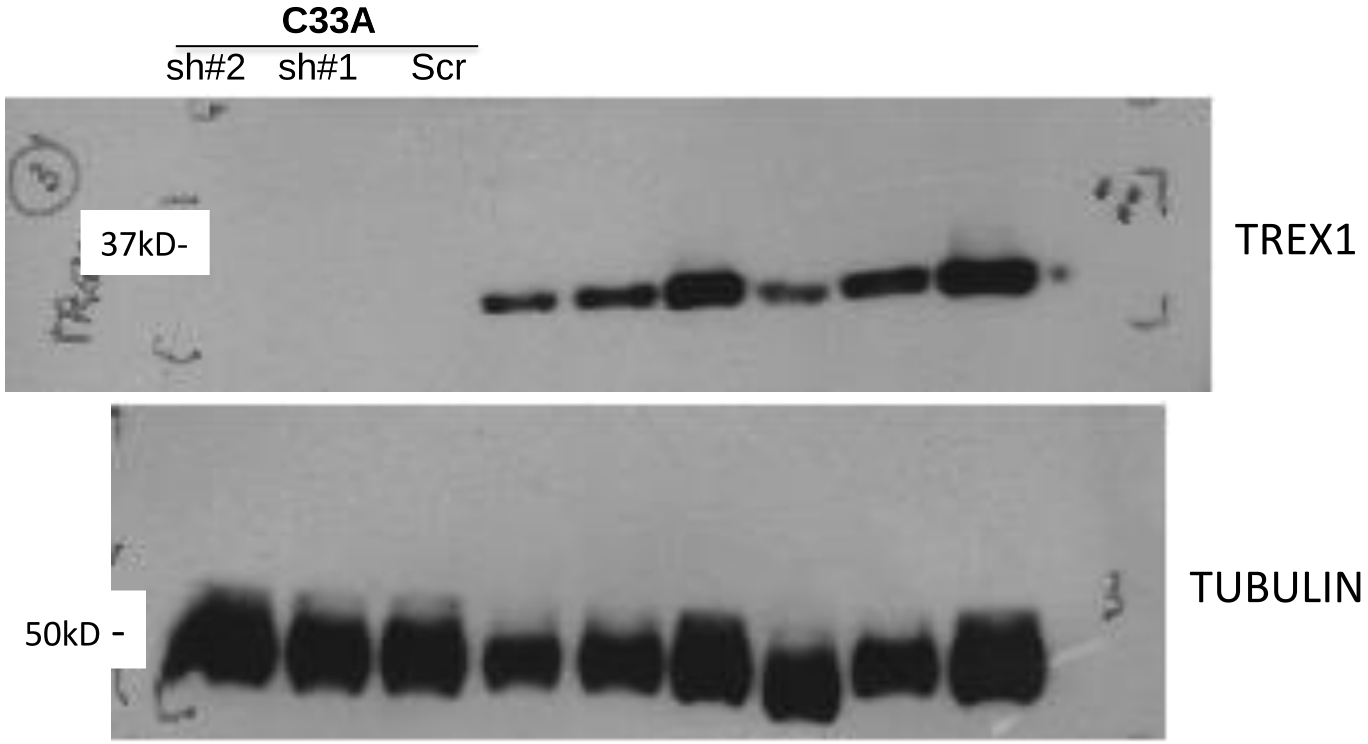

Prati et al. blots for Figure 2

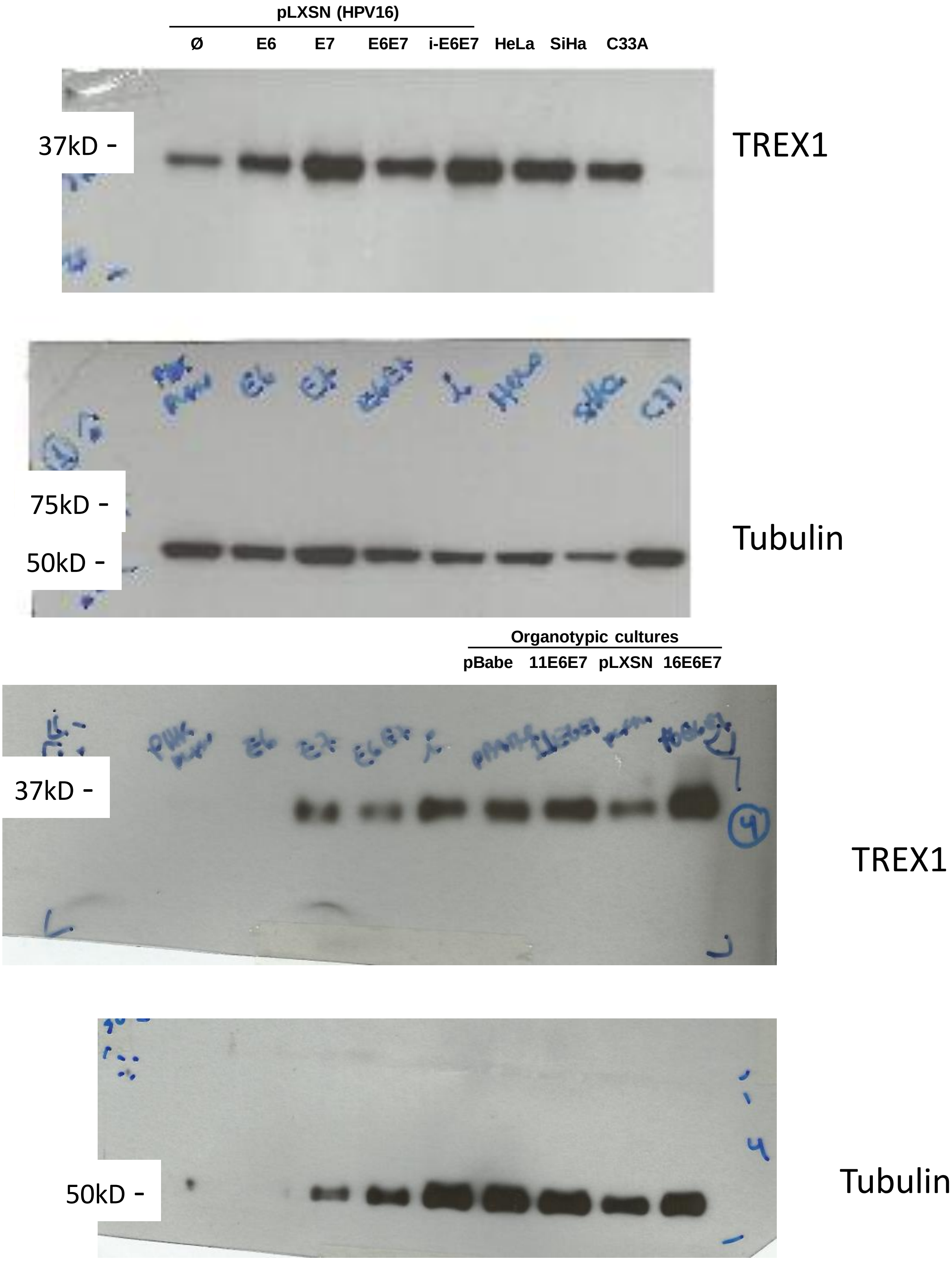

Prati et al. blots for Figure 3

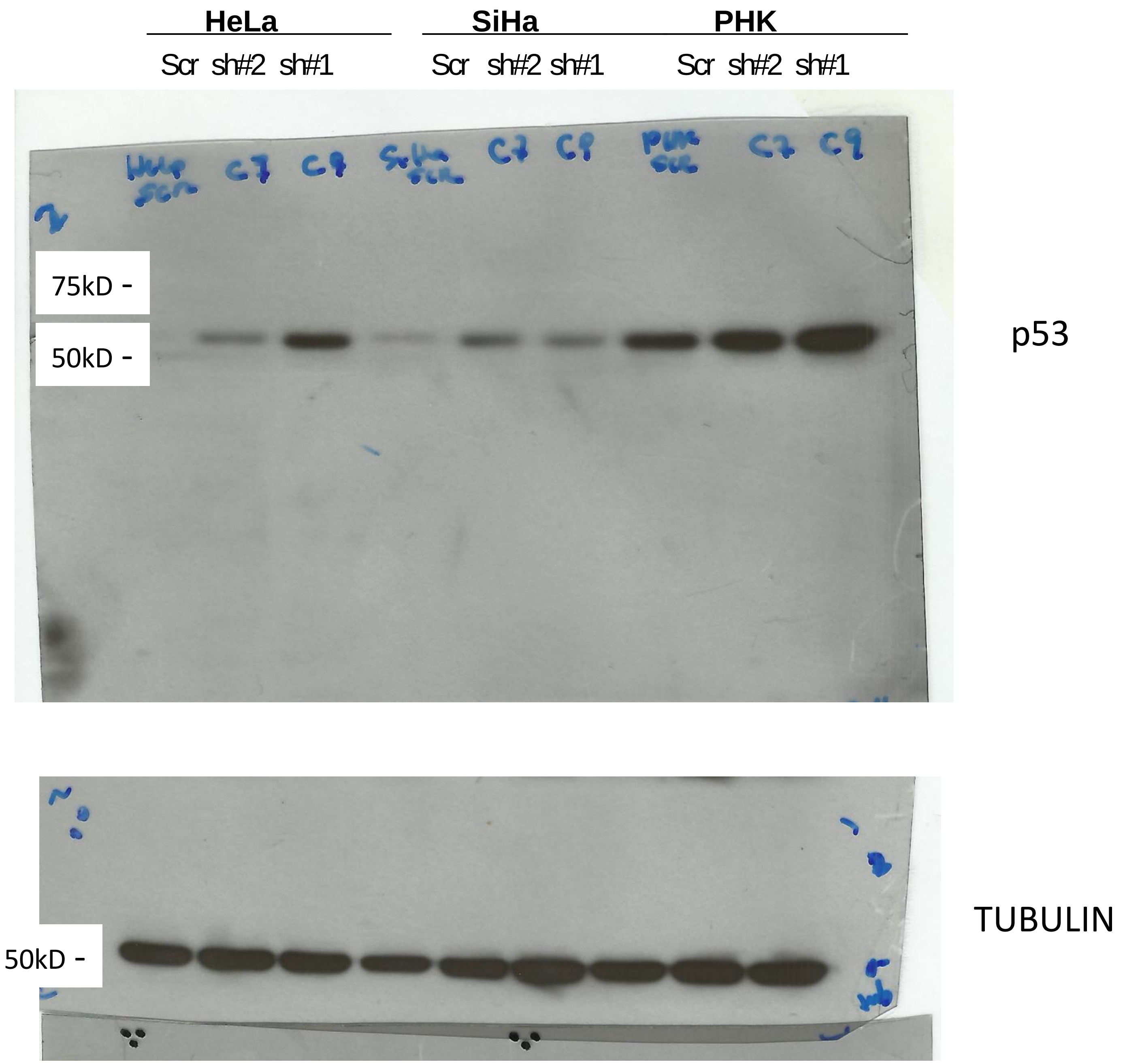

Prati et al. blots for Figure 3

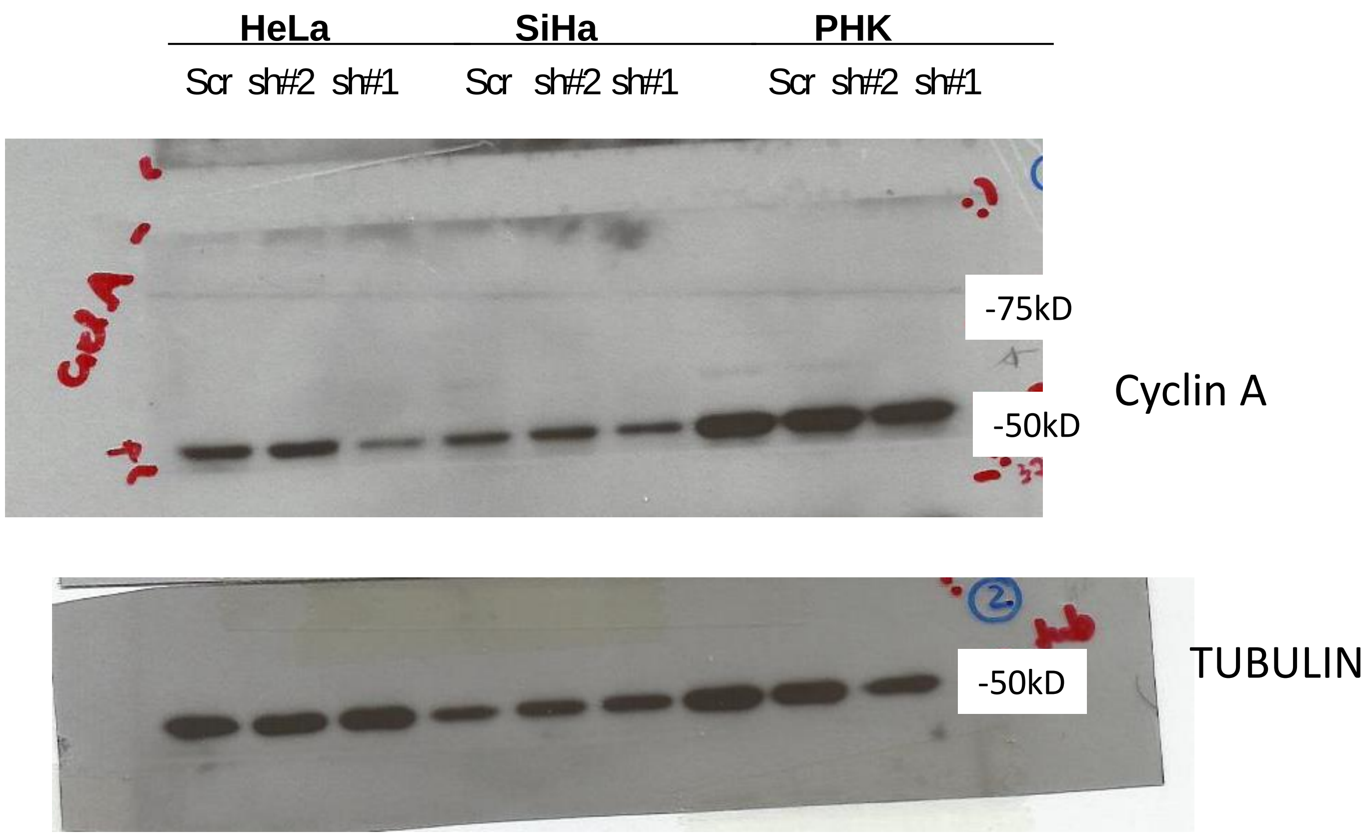

Prati et al. blots for Figure 3

HeLa | **SiHa** | **PHK**

Scr sh#2 sh#1 | Scr sh#2 sh#1 | Scr sh#2 sh#1

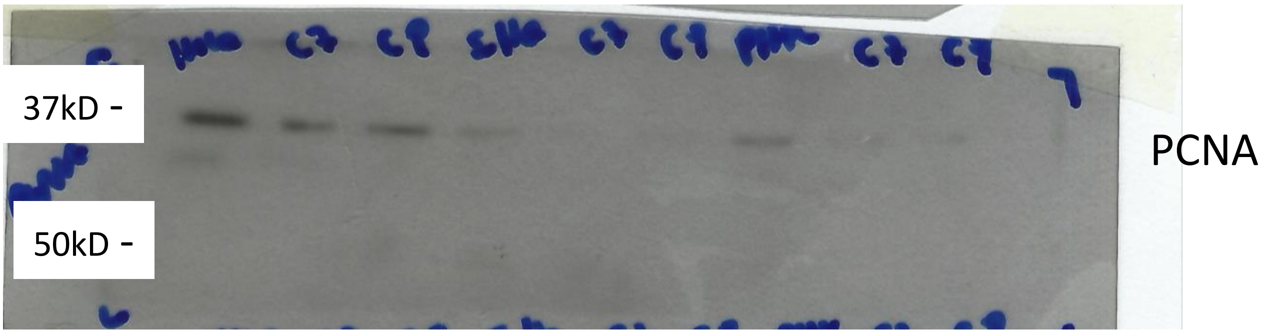

PCNA

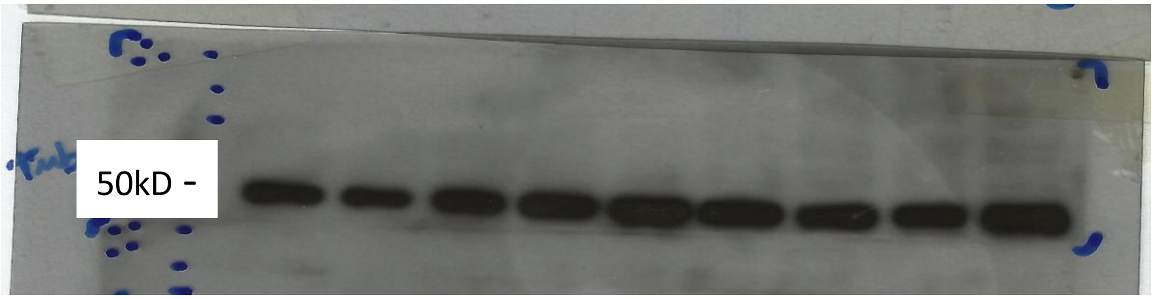

TUBULIN

Prati et al. blots for Figure 3

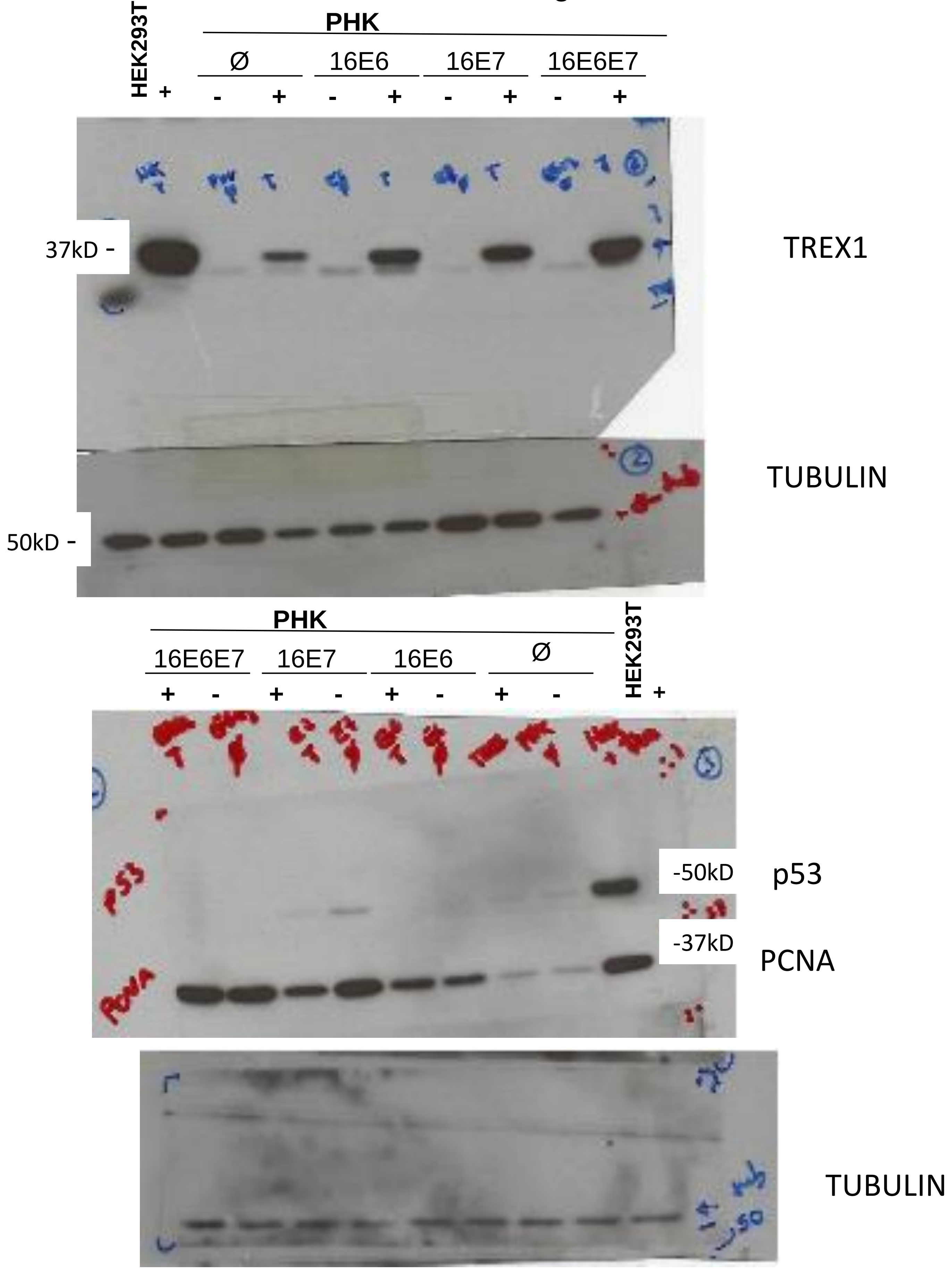

Prati et al. blots for Supplementary figure 2

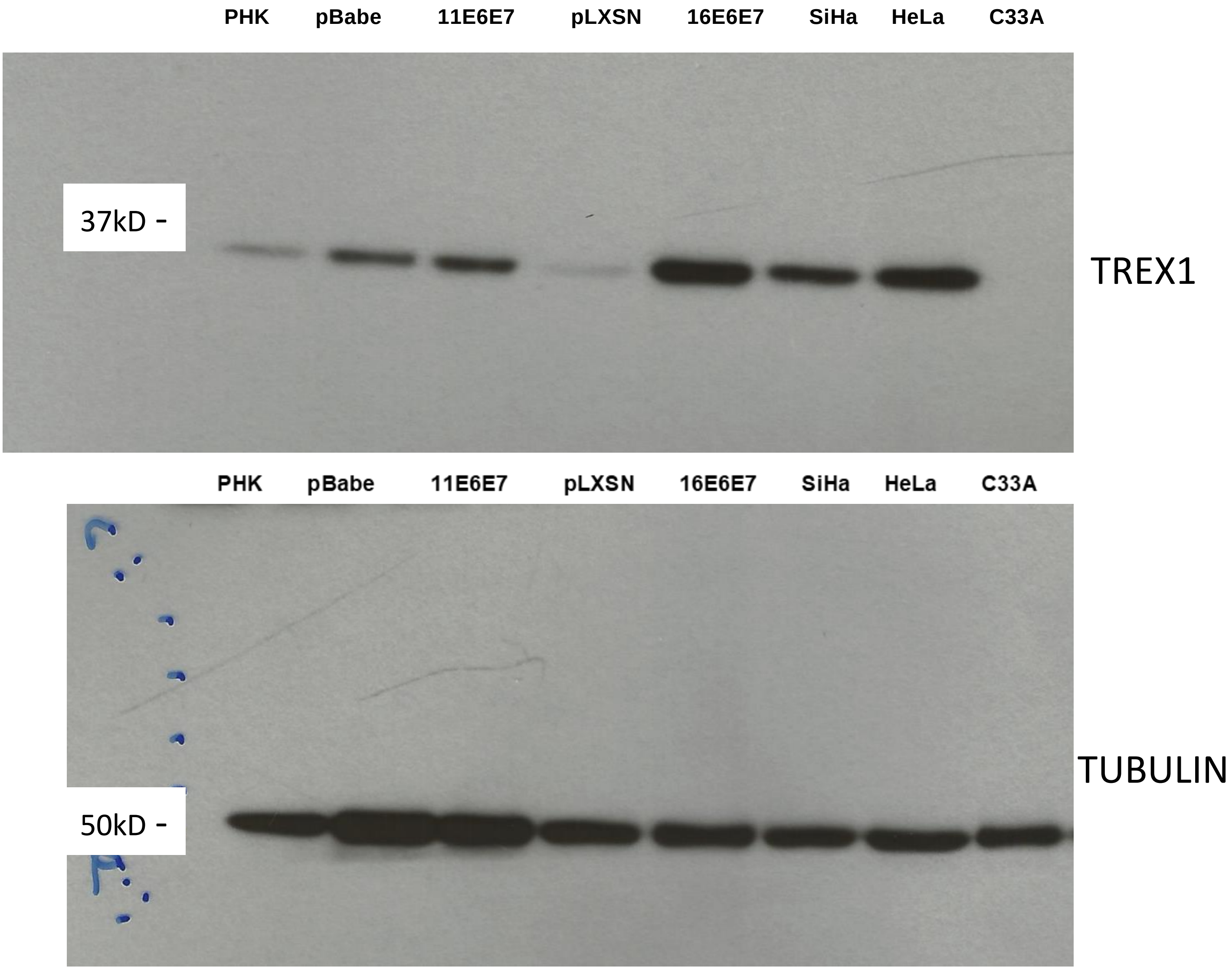

Prati et al. blots for Supplementary figure 2

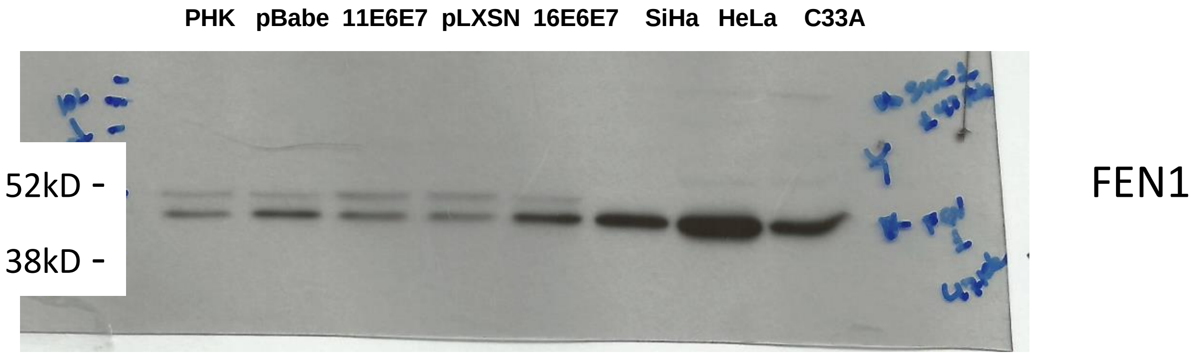

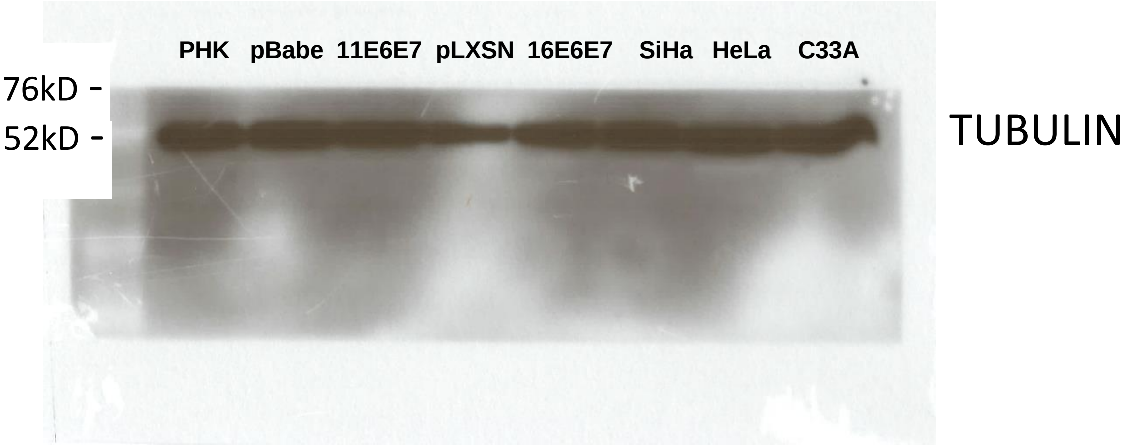

Prati et al. blots for Supplementary figure 2

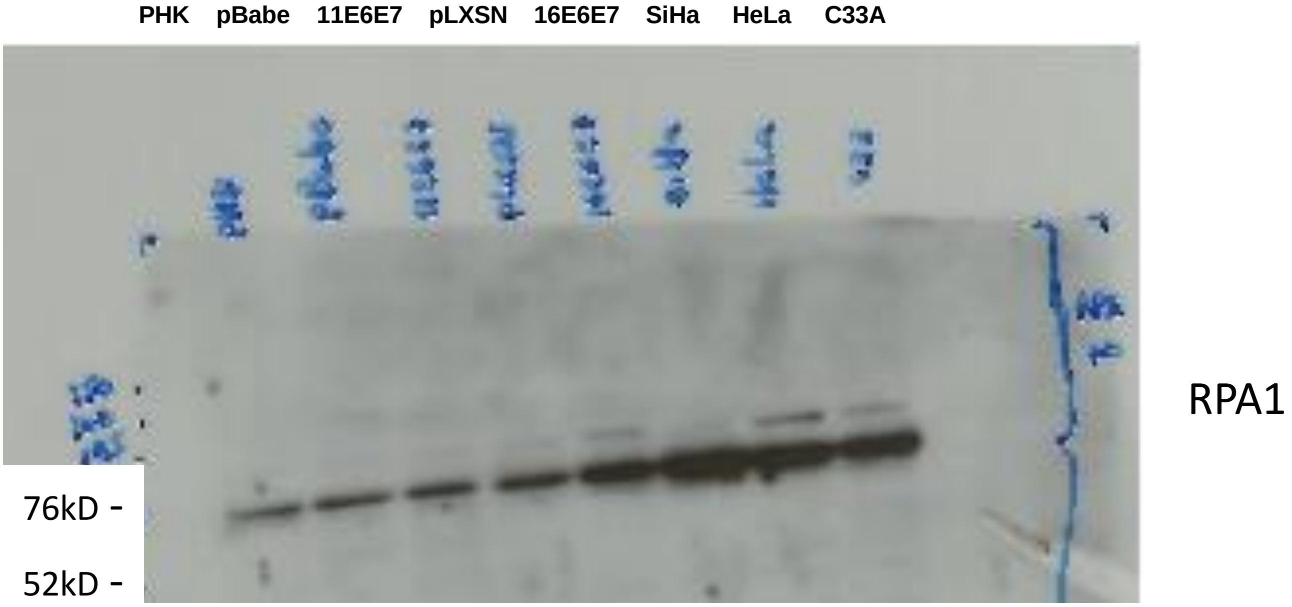

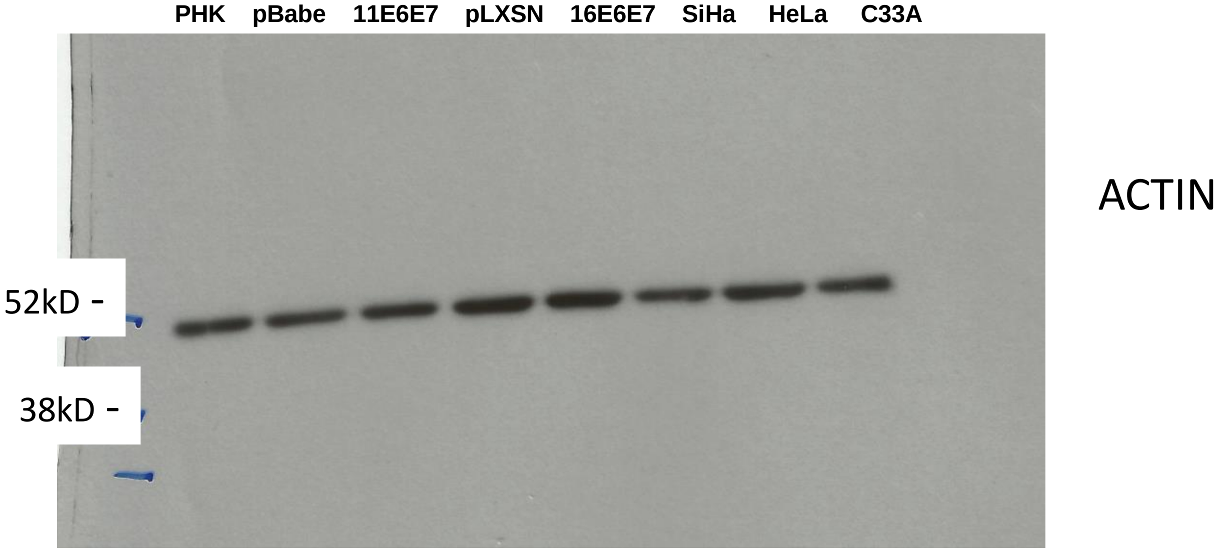

Prati et al. blots for Supplementary figure 2

PHK pBabe 11E6E7 pLXSN 16E6E7 SiHa HeLa C33A

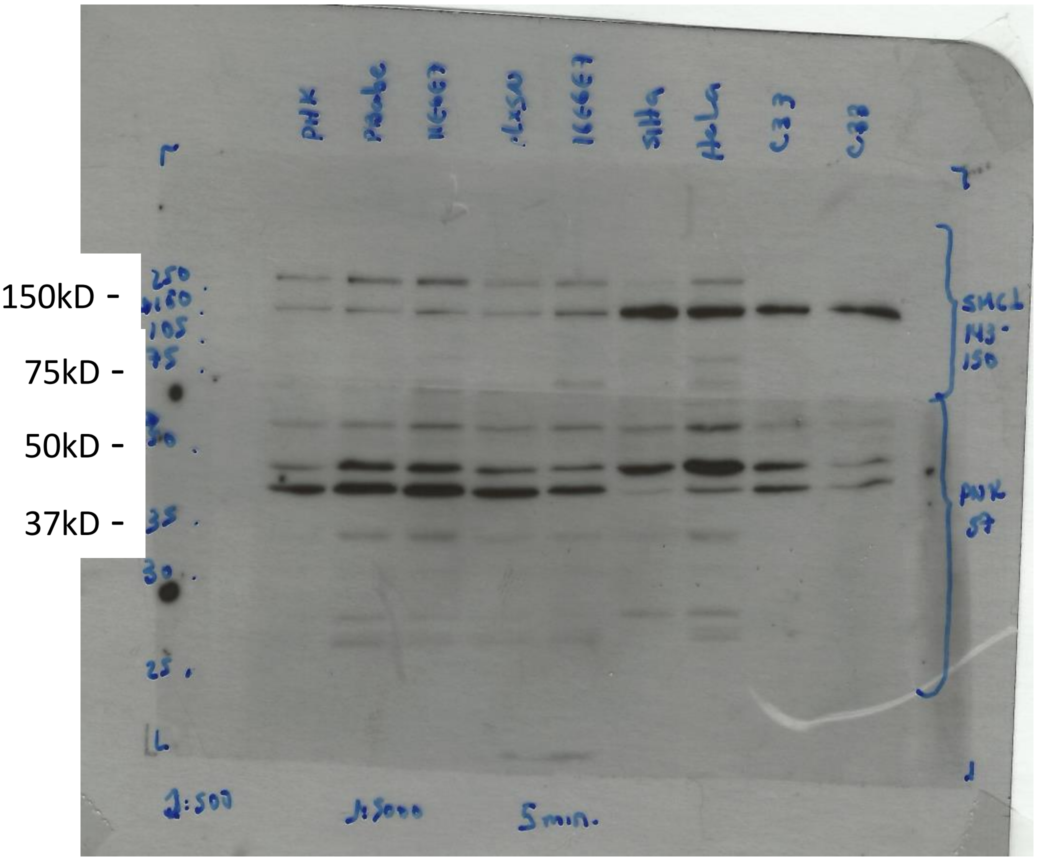

PHK pBabe 11E6E7 pLXSN 16E6E7 SiHa HeLa C33A

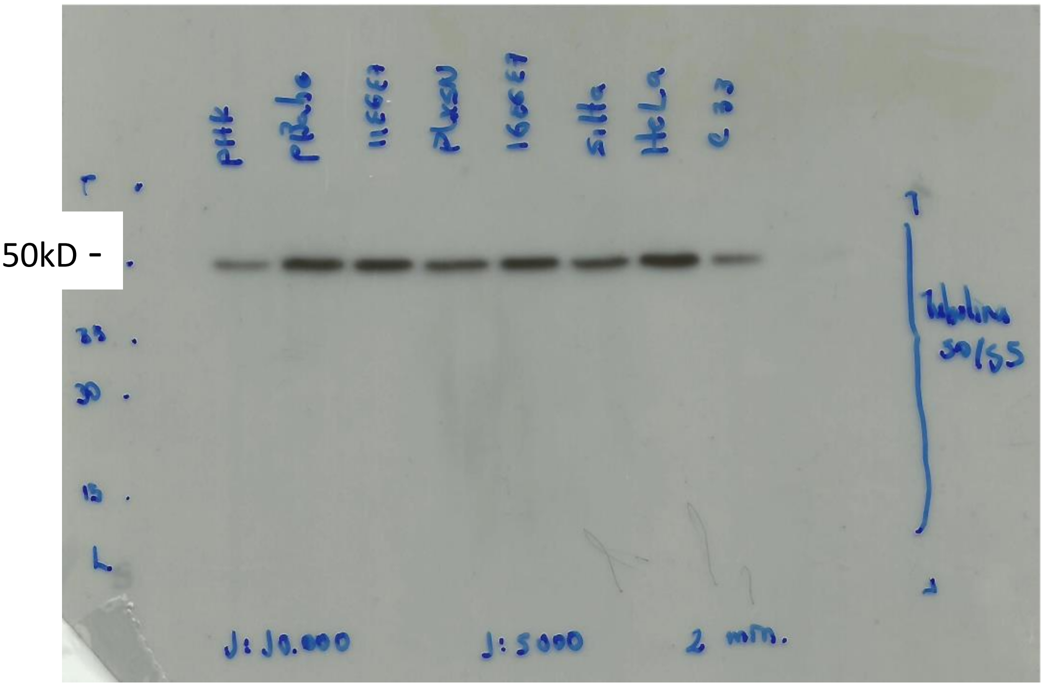

Prati et al. blots for Supplementary figure 2

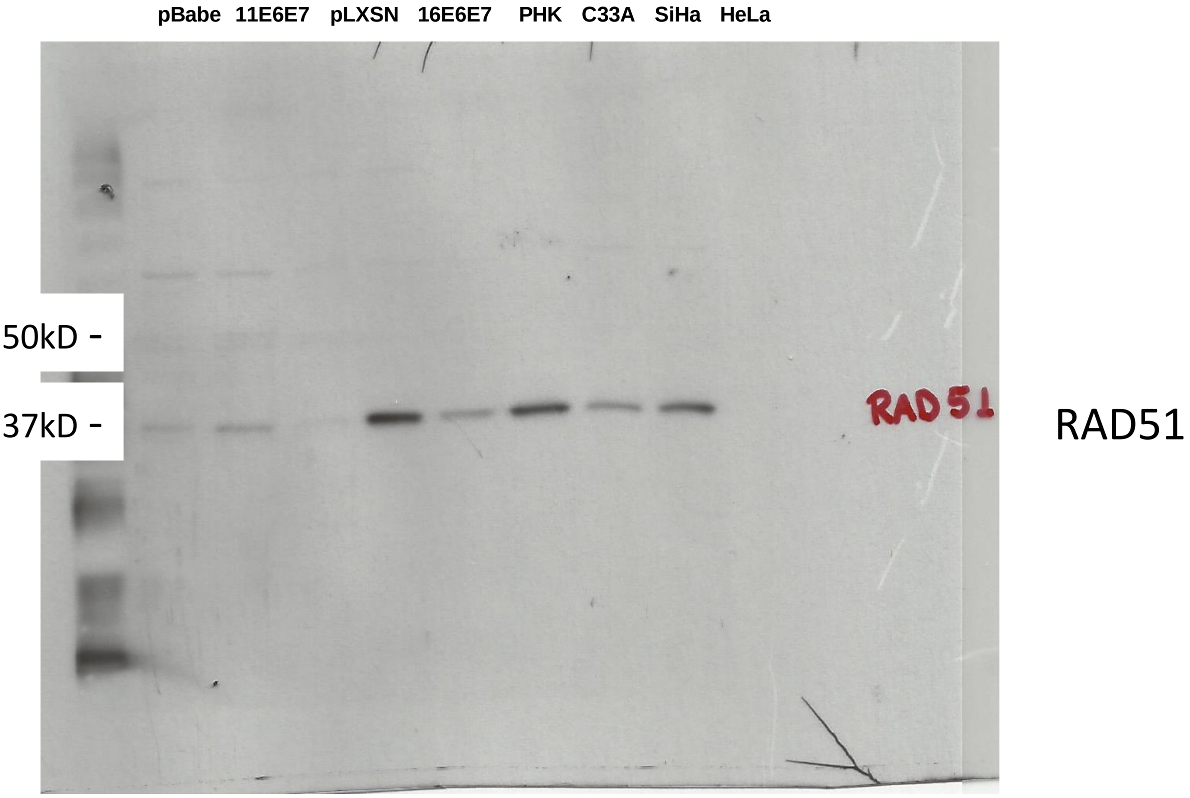

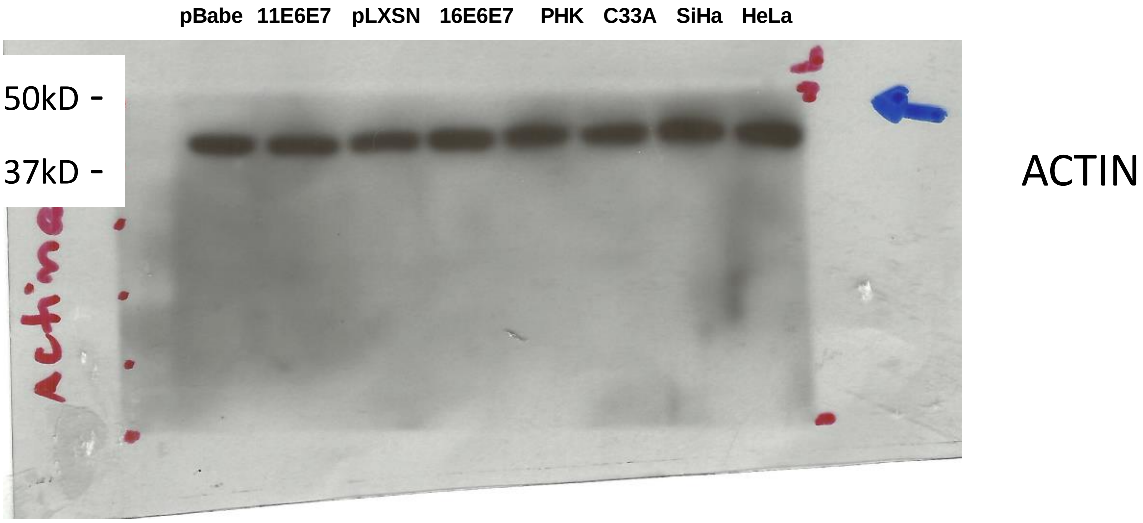

Prati et al. blots for Supplementary figure 2

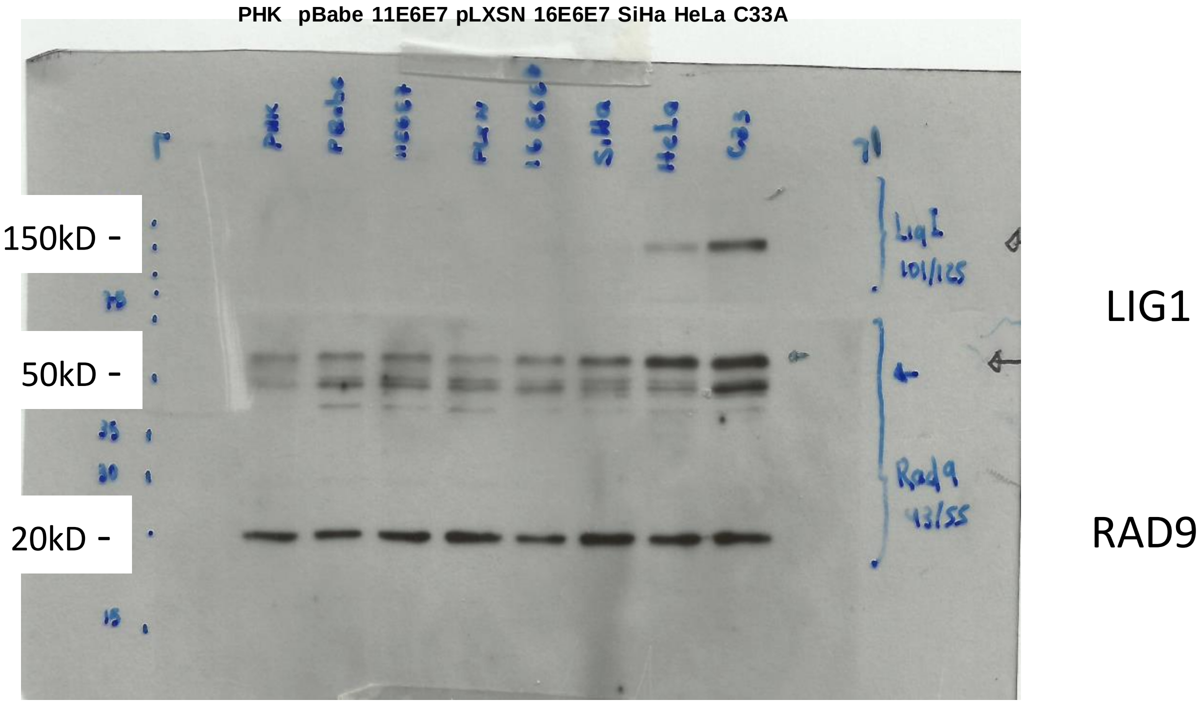

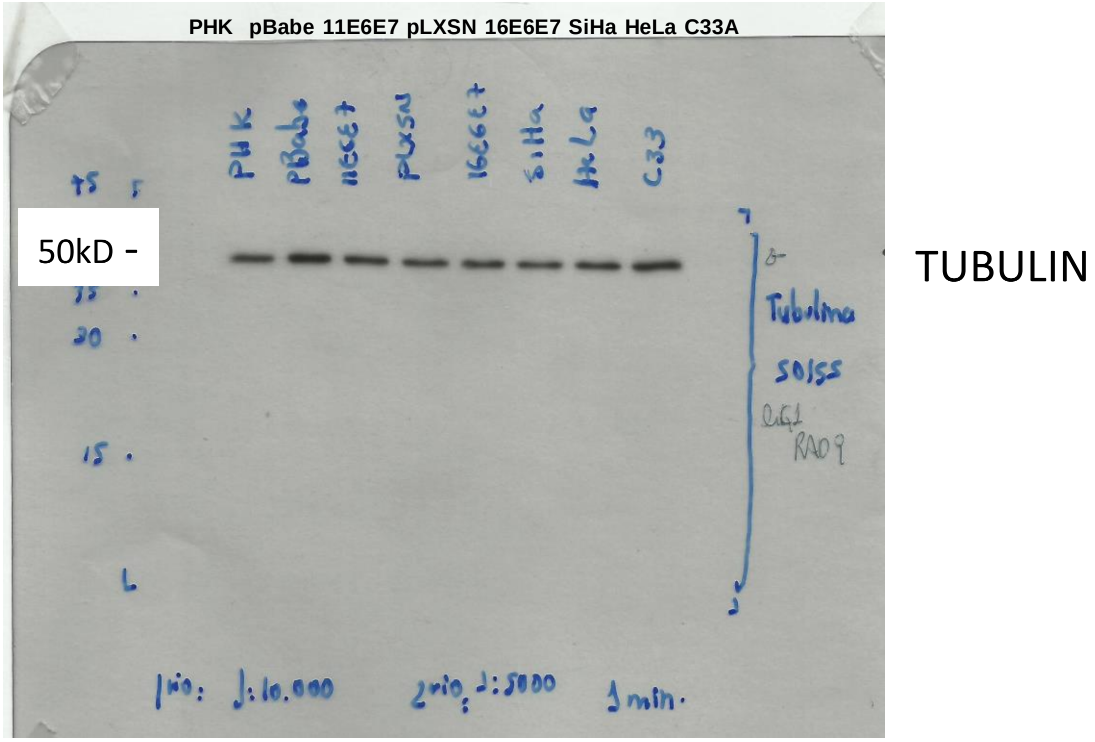

Prati et al. blots for Supplementary figure 2

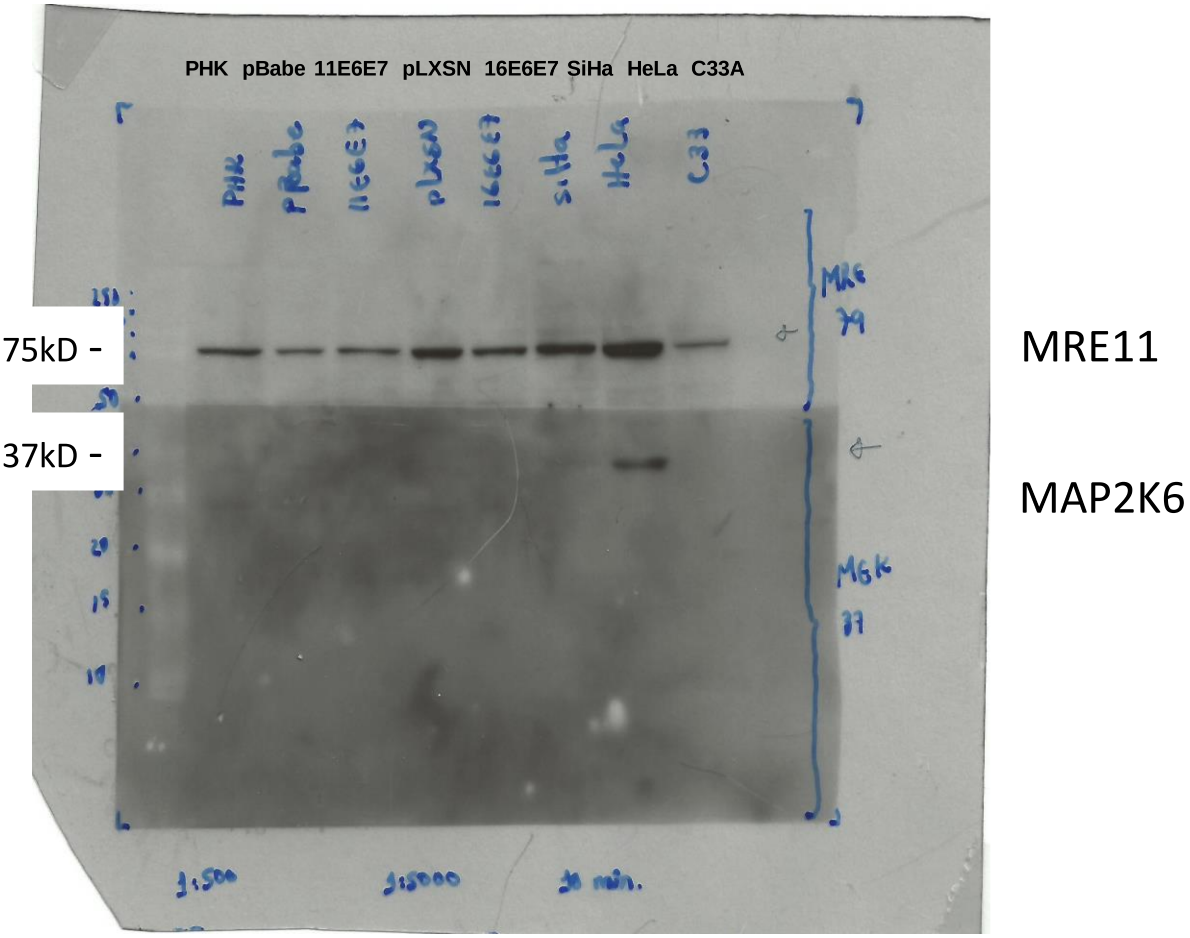

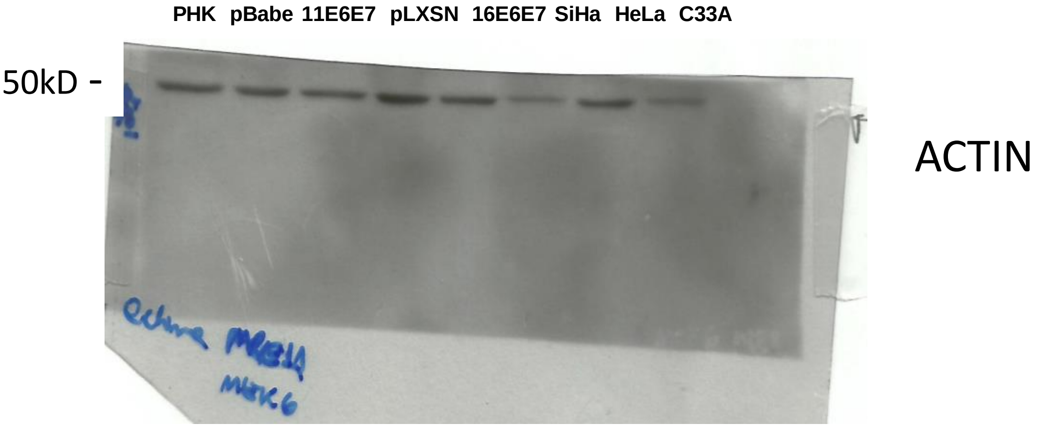

Supplementary figure 2 Prati at al.

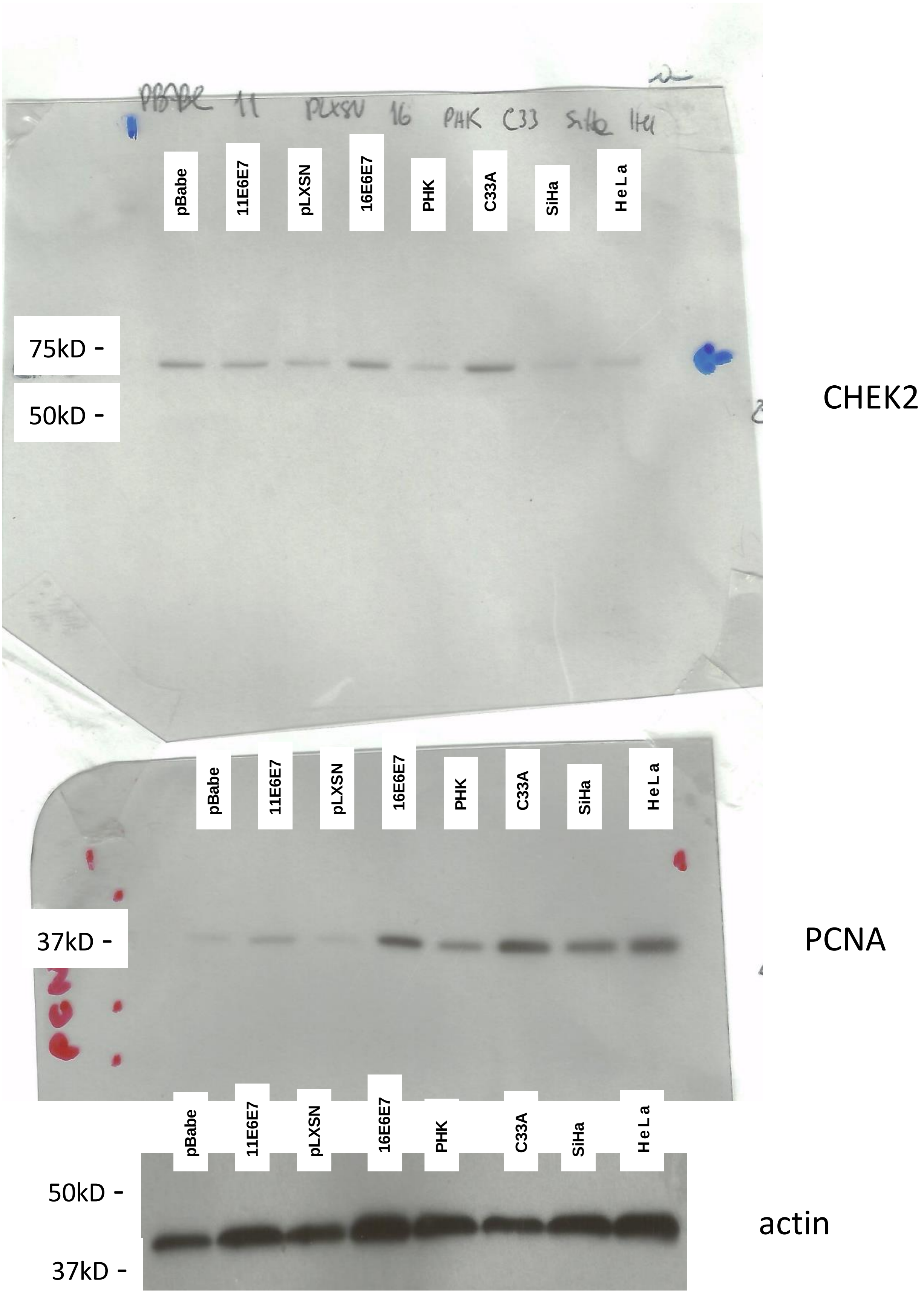

Supplement: Supplementary file 2 — Complete blots [file 41598_2018_37064_MOESM2_ESM.pdf]
